# Spliceosome integrity is defective in the motor neuron diseases ALS and SMA

**DOI:** 10.1002/emmm.201202303

**Published:** 2013-01-25

**Authors:** Hitomi Tsuiji, Yohei Iguchi, Asako Furuya, Ayane Kataoka, Hiroyuki Hatsuta, Naoki Atsuta, Fumiaki Tanaka, Yoshio Hashizume, Hiroyasu Akatsu, Shigeo Murayama, Gen Sobue, Koji Yamanaka

**Affiliations:** 1 Laboratory for Motor Neuron Disease, RIKEN Brain Science InstituteWako, Saitama, Japan; 2 Department of Neurology, Nagoya University Graduate School of MedicineNagoya, Aichi, Japan; 3 Department of Neuropathology, Tokyo Metropolitan Geriatric Hospital and Institute of GerontologyItabashi, Tokyo, Japan; 4 Department of Neuropathology, Fukushimura HospitalToyohashi, Aichi, Japan; 5 Choju Medical Institute, Fukushimura HospitalToyohashi, Aichi, Japan; 6 Japan Science and Technology Agency, CRESTJapan; 7 Brain Science Institute, Saitama UniversitySaitama, Japan; 8 Graduate School of Medicine, Kyoto UniversityKyoto, Japan

**Keywords:** ALS, SMN, snRNA, Spliceosome, TDP-43

## Abstract

Two motor neuron diseases, amyotrophic lateral sclerosis (ALS) and spinal muscular atrophy (SMA), are caused by distinct genes involved in RNA metabolism, TDP-43 and FUS/TLS, and SMN, respectively. However, whether there is a shared defective mechanism in RNA metabolism common to these two diseases remains unclear. Here, we show that TDP-43 and FUS/TLS localize in nuclear Gems through an association with SMN, and that all three proteins function in spliceosome maintenance. We also show that in ALS, Gems are lost, U snRNA levels are up-regulated and spliceosomal U snRNPs abnormally and extensively accumulate in motor neuron nuclei, but not in the temporal lobe of FTLD with TDP-43 pathology. This aberrant accumulation of U snRNAs in ALS motor neurons is in direct contrast to SMA motor neurons, which show reduced amounts of U snRNAs, while both have defects in the spliceosome. These findings indicate that a profound loss of spliceosome integrity is a critical mechanism common to neurodegeneration in ALS and SMA, and may explain cell-type specific vulnerability of motor neurons.

## INTRODUCTION

Defects in RNA metabolism are implicated in many diseases such as cancer, muscular dystrophy and neurodegenerative diseases (Cooper et al, 2009). Those neurodegenerative diseases are characterized by the death of specific types of neurons, and are often caused by mutations in ubiquitously expressed genes. Spinal muscular atrophy (SMA) is caused by deletion or mutations in survival of motor neuron 1 (*SMN1*), amyotrophic lateral sclerosis (ALS) is caused by mutations in superoxide dismutase 1 (*SOD1*), TAR DNA binding protein (*TARDBP*), fused in sarcoma (*FUS/TLS*), or other genes and Huntington's disease is caused by an expansion of CAG repeats in *Huntingtin* (Ule, 2008). ALS is a progressive adult onset neurodegenerative disorder, affecting both the upper and lower motor neurons, whereas SMA is a common genetic cause of death in young children, and affects only lower motor neurons (Andersen & Al-Chalabi, 2011; Burghes & Beattie, 2009; Dion et al, 2009; Lemmens et al, 2010). Since the SMN, TDP-43 (coded by *TARDBP*), and FUS/TLS proteins are all involved in RNA metabolism, a common dysregulation of some aspect of RNA metabolism in motor neurons may underlie these disorders.

Both familial ALS caused by *TARDBP* mutations and sporadic ALS have distinguishing features of clinical pathology in the affected motor neurons, which include the loss of TDP-43 from the nucleus and abnormal formation of cytoplasmic aggregations containing hyper-phosphorylated and ubiquitinated TDP-43 (Arai et al, 2006; Chen-Plotkin et al, 2010; Neumann et al, 2006). Therefore, loss of normal TDP-43 functions and/or gain of toxic cytoplasmic aggregations could be key causative processes of sporadic ALS (Lagier-Tourenne & Cleveland, 2009; Lee et al, 2012). TDP-43 pathology is also seen in a subtype of frontotemporal lobar degeneration (FTLD-TDP), which is a neurodegenerative disease affecting the frontal and temporal lobes (Arai et al, 2006; Chen-Plotkin et al, 2010; Neumann et al, 2006). Therefore, dysfunctions of TDP-43 in distinct neuronal populations can result in different neurodegenerative diseases. However, the mechanisms that underlie neuronal death caused by TDP-43 dysfunctions are not understood for either neurons in spinal cords or in fronto-temporal cortex.

The best characterized function of TDP-43 is in the regulation of pre-mRNA splicing, including the cystic fibrosis transmembrane conductance regulator (Buratti et al, 2001). TDP-43 is believed to regulate many other pre-mRNAs through binding to *cis*-elements in long introns and is also thought to regulate mRNA stability (including its own mRNA; Ayala et al, 2011; Polymenidou et al, 2011). Similarly, FUS/TLS, dominant mutations of which are causative for familial ALS, is a protein possessing multiple functions including regulation in transcription and splicing (Lagier-Tourenne & Cleveland, 2009). Meanwhile, SMN is critical for the assembly of U-rich small nuclear ribonucleoproteins (U snRNPs), which are central components of the spliceosome, and is indispensable to form a nuclear body called Gem in the nucleus (Boulisfane et al, 2011; Burghes & Beattie, 2009; Ebert et al, 2009; Gabanella et al, 2007; Kolb et al, 2007; Talbot & Davies, 2008; Wahl et al, 2009; Wan et al, 2005; Zhang et al, 2008). Therefore, these proteins are involved in RNA metabolism, but whether there are common defects in motor neurons of ALS and SMA patients remains unknown.

In this study, we aimed to determine whether TDP-43 has a similar molecular function to SMN. We also asked whether there are any defects in RNA metabolism common to three distinct neurodegenerative diseases caused by SMN and TDP-43/FUS dysregulation, namely SMA, ALS and FTLD-TDP. We show that TDP-43 and FUS localize in nuclear Gems through association with the SMN complex, and are involved in maintenance of the spliceosome by controlling levels of U snRNAs. We also found abnormal spliceosome accumulation in spinal cord motor neurons from ALS patients but not in the temporal lobe of FTLD-TDP, suggesting that the abnormal splicing caused by collapse of spliceosome integrity is the common process resulting in motor neuron death in ALS and SMA.

## RESULTS

### TDP-43 and FUS interact with the SMN complex in nuclear Gems, and are required for Gem formation

**Figure 1:**
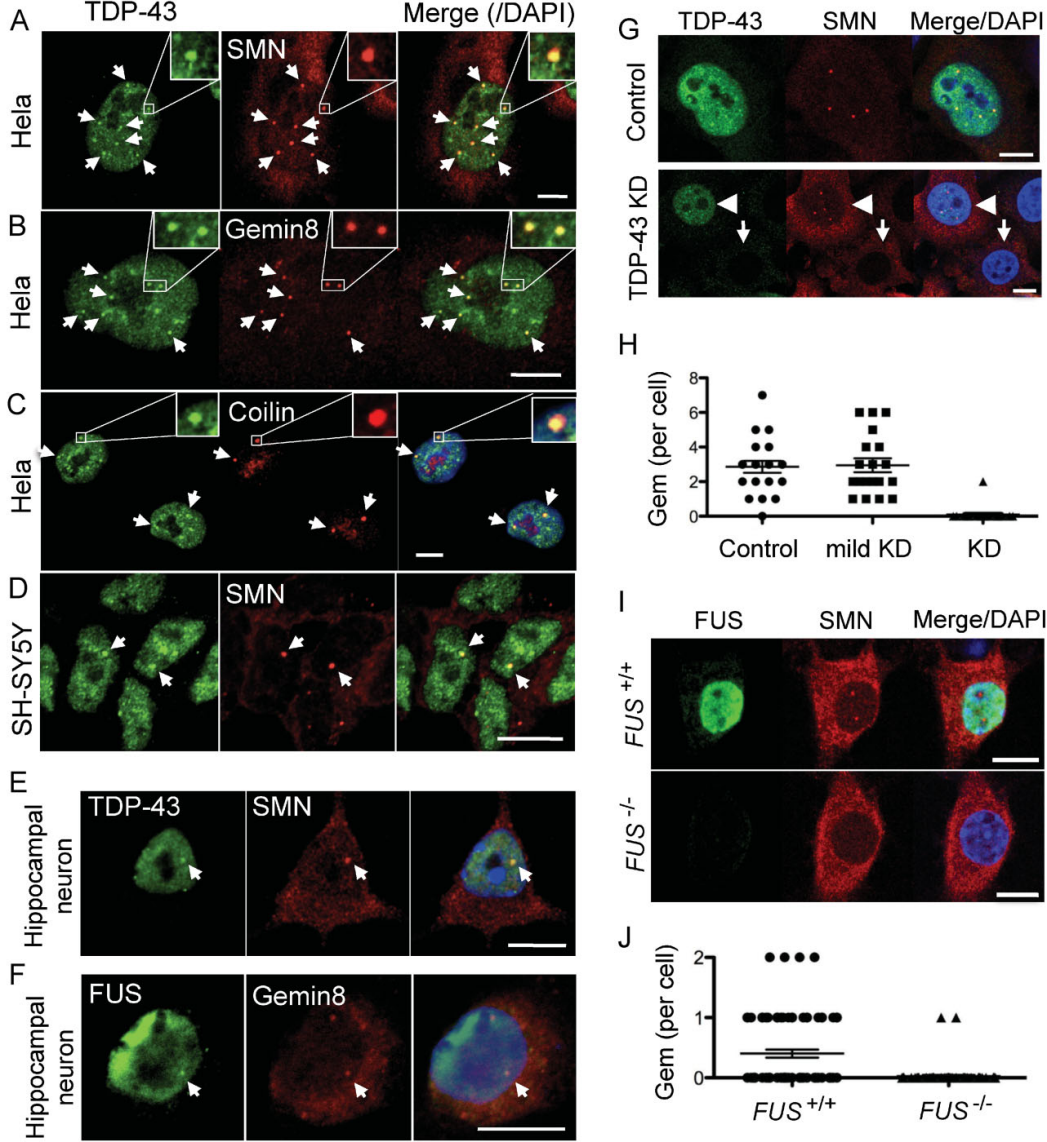
TDP-43 and FUS/TLS interact with the SMN complex in nuclear Gems, and are required for Gem formation **A–C.** Hela cells were immunostained with antibodies against TDP-43 and nuclear domain markers. Magnified images showing colocalization of TDP-43 and nuclear markers (upper right). (**A**,**B**) Costaining of TDP-43 and components of Gems. TDP-43 was extensively concentrated in Gems marked by SMN (**A**, arrows) or Gemin8 (**B**, arrows). (**C**) Costaining of Coilin, a Cajal body marker, and TDP-43. TDP-43 was concentrated in Cajal bodies marked by Coilin (arrows). Bars: 10 µm.**D,E.** TDP-43 localized in Gems of neuronal cell line SH-SY5Y (**D**, arrows) and primary cultured mouse hippocampal neurons (**E**, arrows). Bars: 10 µm.**F.** Costaining of FUS/TLS and Gemin8 in primary cultured mouse hippocampal neurons. FUS/TLS localized in Gem (arrows). Bars: 10 µm.**G.** Hela cells were treated with siRNAs for TDP-43 or control to deplete TDP-43, and immunostained for SMN and TDP-43. Gems are lost in cells with no TDP-43 expression (arrows), whereas they remain in cells with low TDP-43 expression (arrowheads). Bars: 10 µm.**H.** Quantification of Gem numbers in Hela cells treated with siRNAs shown in **G**. Cells with no TDP-43 expression in immunostaining were shown as knockdown (KD), whereas cells with low TDP-43 expression level in immunostaining were shown as mild KD. Means for number of Gems are 2.857 (Control, *n* = 21), 2.947 (mild KD, *n* = 19) and 0.1 (KD, *n* = 20) (Control *vs* KD: *p* < 0.0001).**I.** DIV 21 hippocampal neurons form *FUS*^*−/−*^ mice or littermates were stained for SMN to analyze the requirement of FUS/TLS for Gem formation. Bars: 10 µm.**J.** Quantification of Gems positive for SMN. Means for number of Gems are 0.4026 (*FUS*^+/+^, *n* = 77) and 0.02667 (*FUS*^−/−^, *n* = 75) (*p* < 0.0001).

TDP-43 is an RNA-binding protein that predominantly localizes to the nucleus and regulates pre-mRNA splicing together with SR proteins or with other RNA-binding proteins such as hnRNPA2. The regulation of pre-mRNA is spatially and temporally controlled by splicing factors such as SR proteins that concentrate in nuclear speckles (Kumaran et al, 2008), suggesting that TDP-43 localizes to nuclear speckles as well as nucleoplasm. However, sub-nuclear localization of TDP-43 has been debated (Casafont et al, 2009; Fiesel et al, 2010; Shan et al, 2010; Wang et al, 2002). Our detailed analysis of sub-nuclear TDP-43 distribution revealed that TDP-43 was concentrated in Gems, which are marked by survival of motor neuron (SMN; Fig 1A, arrows) or Gemin8 (Fig 1B, arrows); Cajal bodies, which are marked by Coilin (Fig 1C, arrows); and paraspeckles, which are marked by p54^nrb^ (Supporting Information Fig S1A, arrows). TDP-43 was partially overlapped with the SR protein SRSF2/SC35 in nuclear speckles (Supporting Information Fig S1B, arrows), and was not concentrated in PML bodies, the nucleolus, or SAM bodies (Supporting Information Fig S1C–E). TDP-43 was also localized to Gems in the neuronal cell line SH-SY5Y and in primary cultured neurons from mouse hippocampus (Fig 1D and E). FUS/TLS, an ALS-causative protein, was also localized to Gems in Hela cells (data not shown) and in primary cultured neurons from mouse hippocampus (Fig 1F). This is consistent given the known interaction of TDP-43 and FUS. Therefore, it is evident that both TDP-43 and FUS, RNA binding proteins of which mutations cause ALS, colocalize to Gems along with SMN.

To test whether TDP-43 is required for Gem formation, Hela cells were treated with either siRNAs that targeted TDP-43 or with control siRNAs, and were then immunostained for SMN. By Western blot analysis, we determined that TDP-43 protein levels in siRNA-treated cells were downregulated to about 10% of the control levels (Supporting Information Fig S2B), but the percentage of downregulation varied among cells (Supporting Information Fig S2A). Gems were abolished in cells that had no detectable TDP-43 expression (indicated as KD), but remained in cells that had low TDP-43 expression levels (indicated as mild KD; Fig 1G and H and Supporting Information Fig S2A). To assess the importance of FUS for Gem formation, we utilized FUS knockout mice. Gems are lost in primary cultured hippocampus neurons (at 21 days *in vitro*) from FUS knockout mice (Fig 1I and J), whereas total SMN protein levels are unaffected in FUS knockout cells (Supporting Information Fig S2C). These results indicate that TDP-43, FUS and SMN (Ebert et al, 2009) are all required for formation of nuclear Gems.

**Figure 2:**
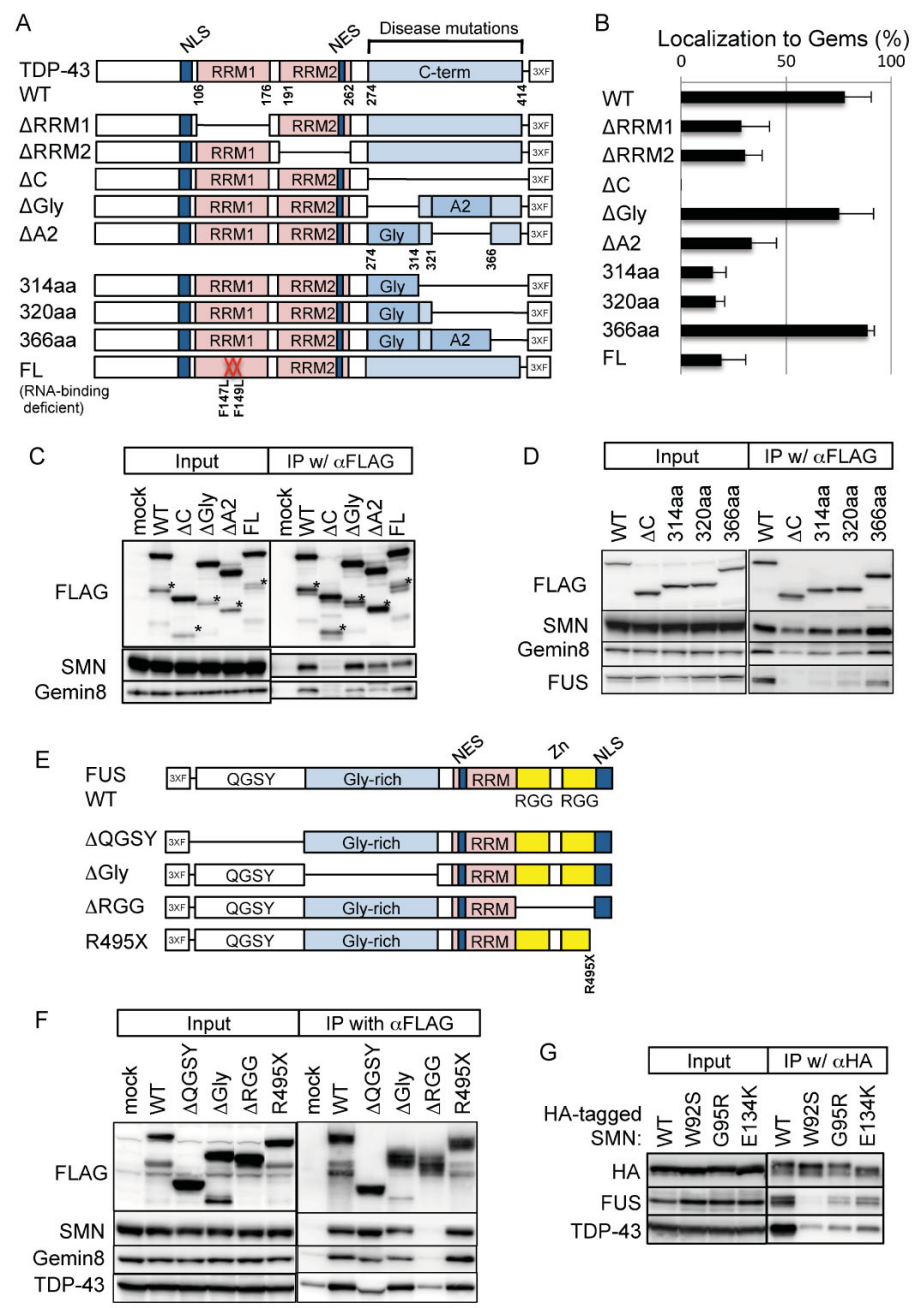
Determination of domains required for association of TDP-43, FUS/TLS and SMN complex **A.** A schematic diagram of C-terminal 3XFLAG-tagged expression constructs for TDP-43 used in this study.**B.** The latter half of the C-terminal glycine-rich region of TDP-43 was important for the proper localization to Gems. Hela cells were transfected with TDP-43-3XFLAG or indicated mutants, and stained with anti-SMN and anti-FLAG antibodies. Co-localization of TDP-43 and SMN was assessed by confocal microscope, numbers of TDP-43-positive Gems and -negative Gems were counted. More than 100 Gems were counted for each construct, and the localization to Gem (%) was defined as TDP-43-postive Gems per total Gems (%). To eliminate variation in the number of Gems per nucleus, cloned Hela cells were used. The average and error bars from three independent experiments were plotted.**C,D.** The SMN/Gemin8/FUS interactions with TDP-43 were dependent on the TDP-43 C-terminus. TDP-43-3xFLAG mutants were expressed in Hela cells, and the TDP-43 interacting proteins were immunoprecipitated using an anti-FLAG antibody and identified by Western blot analysis using the specific antibodies as indicated. Asterisks indicate degraded FLAG-tagged TDP-43 proteins.**E.** A schematic diagram of N-terminal 3XFLAG-tagged expression constructs for FUS/TLS used in this study.**F.** 3xFLAG-FUS/TLS mutants were expressed in Hela cells, and the FUS/TLS interacting proteins were immunoprecipitated using an anti-FLAG antibody and identified by Western blot analysis using the specific antibodies as indicated.**G.** Mutations in Tudor domain of SMN1 decreased association of TDP-43 and FUS/TLS. HA-tagged human SMN1 and its mutants were expressed in Hela cells, and SMN interacting proteins were immunoprecipitated using an anti-HA antibody and identified by Western blot analysis using the specific antibodies as indicated.

To identify the region within TDP-43 that is required for localization to Gems, we performed domain analysis using deletion mutants of TDP-43 (Fig 2A). Our analysis revealed that the C-terminus of TDP-43, where several known ALS-linked mutations are located, was responsible for the proper localization to Gems (Fig 2B and Supporting Information Fig S3). More specifically, amino acids 321–366 of the C-terminus, which interact with hnRNP A2 (D'Ambrogio et al, 2009), were important for localization to Gems (Fig 2B). Furthermore, the RNA-binding activity of TDP-43 is partially required, as both RNA-recognition motif 1 (RRM1) deletion mutants and F147L/F149L double point mutants had reduced localizations to Gems (Fig 2B). These results indicate that protein–protein interactions mediated by the latter half of the C-terminal region and the RNA binding activity play critical roles in the proper localization of TDP-43 to Gems.

We next asked whether TDP-43 or FUS/TLS associate with protein complexes that contain SMN. An interaction of TDP-43 with endogenous SMN/Gemin8 and other spliceosome components was confirmed by immunoprecipitation, and this interaction was dependent on both the C-terminus of TDP-43 and the RNA binding activity of TDP-43 (Fig 2C and D). Strikingly, these were the same regions that were required for localization of TDP-43 to Gems (Fig 2B), indicating that TDP-43 and SMN components may be recruited to Gems together. We also assessed which regions of FUS/TLS were important for interactions with SMN containing protein complexes. We found that the C-terminal RGG rich region of FUS/TLS was important for the interaction with SMN complexes and TDP-43 (Fig 2E and F). Furthermore, while FUS/TLS and TDP-43 proteins interacted with overexpressed HA-tagged SMN, they did not interact with SMN proteins that contained mutations in the tudor domain (W92S, G95R and E134K). These mutations are known to reduce affinity to RG repeats of Sm proteins and cause SMA (Tripsianes et al, 2011; Fig 2G). These results indicate that SMN associates with FUS/TLS and TDP-43 through an interaction between the tudor domain of SMN and the RGG domain of FUS. Therefore, three proteins implicated in motor neuron disease, TDP-43, FUS/TLS and SMN, interact with each other.

### Loss of gems in motor neurons from ALS patients

**Figure 3:**
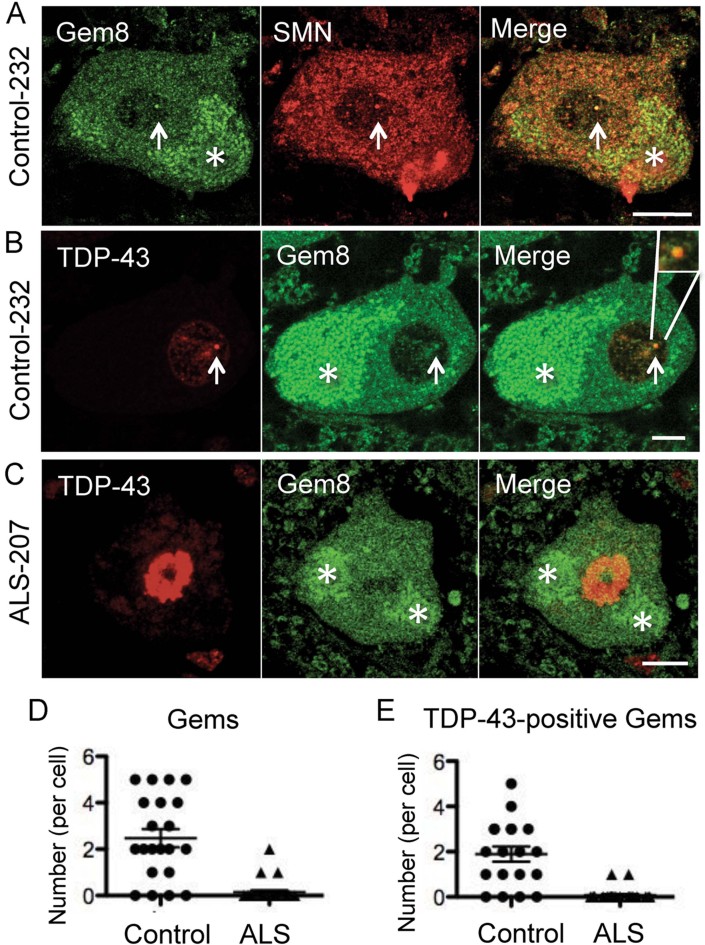
TDP-43-positive Gems are decreased in motor neurons from ALS patients Immunostaining of Gems in human spinal cord motor neurons. Paraffin-fixed post-mortem spinal cords from patients with neurological diseases other than ALS were analysed under a confocal microscope for the presence of Gems with antibodies against SMN and Gemin8 (Gem8) (arrows). Note that autofluorescence derived from lipofuscin was observed in the cytoplasm (asterisks). Bars: 10 µm.Coimmunostaining of TDP-43 and SMN indicating the presence of TDP-43-positive Gems in human spinal cord motor neurons (arrows). Bars: 10 µm.Coimmunostaining of TDP-43 and Gemin8 in remaining motor neurons of ALS spinal cords. TDP-43 is localized in the nucleus. Bars: 10 µm.Nuclear foci with a significant concentration with Gemin8 and SMN were defined as Gem, and numbers of Gems in motor neurons from three control patients (*N* = 21) or three ALS patients (*N* = 25) were counted. Means are 2.476 and 0.08, respectively (*p* < 0.0001).Nuclear foci with a significant concentration with Gemin8 as determined in (**D**) were defined as Gem, and numbers of TDP-43-positive Gems in motor neurons of spinal cords with control disease (*N* = 19) or ALS (*N* = 18). Means are 1.895 and 0.056, respectively (*p* < 0.0001).

Considering that Gems in human motor neurons differentiated from SMA patient-derived iPS cells are decreased compared with control (Ebert et al, 2009), it is very important to ask whether Gems are present in motor neurons of human spinal cords, and whether Gems are TDP-43-immunopositive. Post-mortem lumbar spinal cord tissues from ALS and non-ALS patients were stained. The accumulations that we observed of SMN and Gemin8, the two principal components of Gems in nuclei, indicated the presence of Gems in motor neurons from non-ALS human spinal cords (Fig 3A, arrows). Moreover, we found that TDP-43 localized to Gems in human motor neurons (Fig 3B, arrows). We quantified the number of Gems by double-staining for SMN and Gemin8, resulting in an average of 2.5 Gems per spinal cord motor neuron in control patients (Fig 3D). The average number of TDP-43-immunopositive Gems was 1.9 per spinal cord motor neuron in control patients (Fig 3E). Intriguingly, in motor neurons from ALS patients, with abnormal TDP-43 accumulation, Gemin8 was distributed uniformly throughout the nucleus and cytoplasm (Fig 3C). The quantification of numbers of Gems and TDP-43-positive Gems (0.08 and 0.06, respectively) revealed a significant loss of Gems in motor neurons from ALS patients (Fig 3D and E). The loss of Gems is also a feature of motor neurons from SMA patients, implicating the importance of Gem formation for motor neurons.

### Alteration of U snRNA levels with TDP-43 depletion

**Figure 4:**
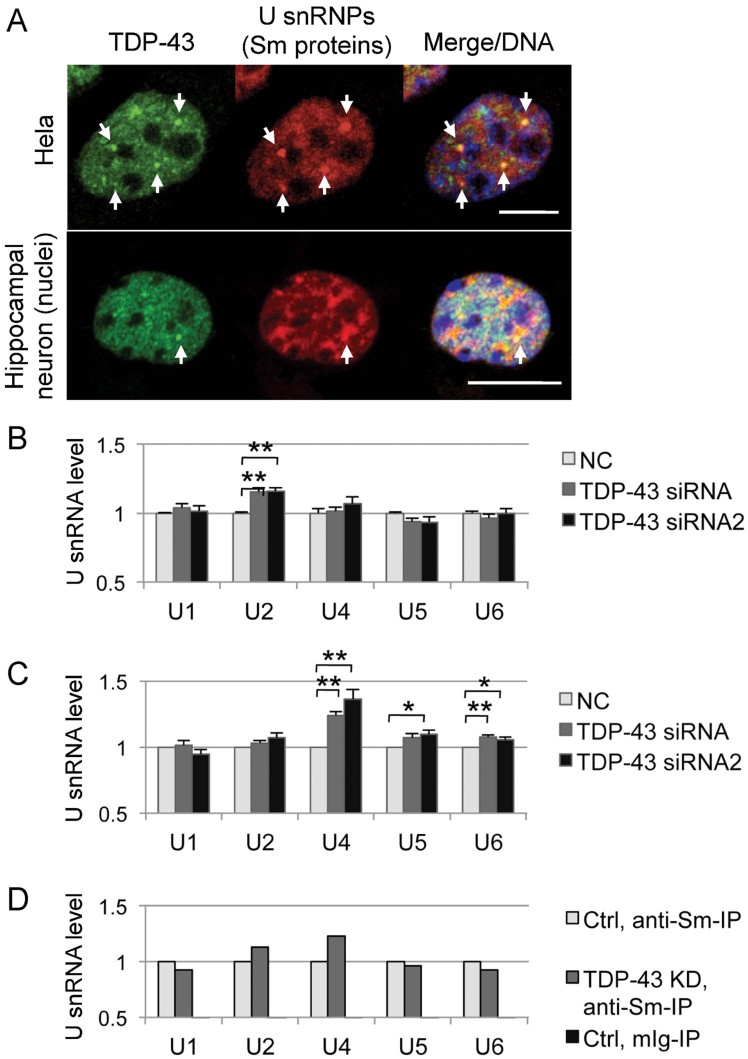
TDP-43 is associated with U snRNPs and is required for maintaining proper expression levels of U snRNAs **A.** TDP-43 colocalized with U snRNPs that were marked with an anti-dimethylated Sm proteins antibody (Y12) in the nuclei of Hela cells and primary cultured mouse hippocampal neurons. Note that U snRNPs and TDP-43 were concentrated in the same nuclear bodies (arrows). Bar: 10 µm.**B,C.** U snRNA levels in Hela cells (**B**) or SH-SY5Y cells (**C**) treated with siRNAs for TDP-43 or control were determined by quantitative RT-PCR. Average from three independent experiments with transfections performed in triplicate were plotted (Bars: standard errors, *p < 0.05, **p < 0.01, Student's *t*-test).**D.** Mature U snRNP-associated U snRNA levels in Hela cells treated with siRNAs for TDP-43 or control. U snRNA levels were determined by quantitative RT-PCR from the RNAs isolated from mature U snRNP complex which was immunopurified using anti-Sm proteins antibody (Y12) as described in Materials and Methods.

The association and localization of TDP-43, FUS/TLS and SMN in Gems imply a functional convergence of three proteins. SMN is well known to assist in assembly of U snRNPs, which is central to splicing, in the cytoplasm and to recruit U snRNPs into the nucleus (Pellizzoni et al, 2002). In SMA mice and SMN-depleted cells, the levels of U snRNAs and components of U snRNPs are unbalanced, resulting in aberrant splicing (Gabanella et al, 2007; Zhang et al, 2008). Therefore, we hypothesized that TDP-43 might have a function in U snRNP biogenesis and alterations in U snRNPs may be also responsible for ALS. To test this hypothesis, we first analysed if TDP-43 associated with U snRNPs. TDP-43 distribution was similar to U snRNPs, which were marked by the anti-dimethylated Sm proteins antibody (Y12), and both TDP-43 and U snRNPs were concentrated to same nuclear bodies in Hela cells and primary cultured mouse hippocampal neurons (Fig 4A, arrows), suggesting the interaction of TDP-43 and snRNPs. Since C-terminus of TDP-43 is required for TDP-43-containing foci in nuclei (Fig 2B, Supporting Information Fig S3C), we identified proteins interacting with C-terminus of TDP-43. Comparison of proteins immunoprecipitated with wild type FLAG-tagged TDP-43 or deletion mutant of C-terminal domain, followed by LC–MS/MS, revealed many proteins associated with a TDP-43 C-terminus including U snRNP components such as PRPF3 (Supporting Information Fig S4A and B). The association of TDP-43 with U snRNP components PRPF3 and U1-70K was confirmed by IP-Western blotting (Supporting Information Fig S4C). To investigate the association between U snRNPs and TDP-43, U snRNPs were immunoprecipitated with the anti-Sm proteins antibody (Y12) (Supporting Information Fig S4D). Subsequent immunoblotting confirmed that TDP-43 was co-immunoprecipitated with U snRNPs although at a relatively low level. These results suggest a possible involvement of TDP-43 in maintaining the integrity of U snRNPs.

Next, we measured levels of U-rich small nuclear RNAs (U snRNAs), the major components of U snRNPs, following TDP-43 knockdown (Gabanella et al, 2007; Zhang et al, 2008). Surprisingly, despite the lack of Gems, up-regulation of U snRNAs was observed in TDP-43 depleted cells. U2 snRNA levels were up-regulated in TDP-43 depleted Hela cells (Fig 4B), and U4, U5 and U6 snRNAs were up-regulated in TDP-43-depleted neuronal SH-SY5Y cells (Fig 4C). These results show that the dysfunction of TDP-43 causes misregulation of U snRNAs, although misregulated U snRNAs were different between these neuronal and non-neuronal cells. This is intriguing, because SMN-dysfunction causes cell-type specific misregulation of repertoires of U snRNAs, with decrease of distinct subsets of U snRNAs in different cell types (Gabanella et al, 2007; Zhang et al, 2008). We also measured levels of U snRNAs that are associated with mature U snRNPs in the nuclei of TDP-43 depleted cells, by immunopurification of U snRNPs from nuclei with the anti-Sm proteins antibody (Y12). The pattern of changes seen in U snRNA levels from mature U snRNP fraction (Fig 4D) was similar to that seen when U snRNAs were extracted from whole cells (Fig 4B). Therefore, the levels of U snRNAs associated with Sm proteins were up-regulated in the nuclei of TDP-43 depleted cells. Taken together, these data indicated that TDP-43 is important for maintaining the proper levels of U snRNAs.

### U snRNA levels are aberrantly upregulated in ALS, but not in FTLD

**Figure 5:**
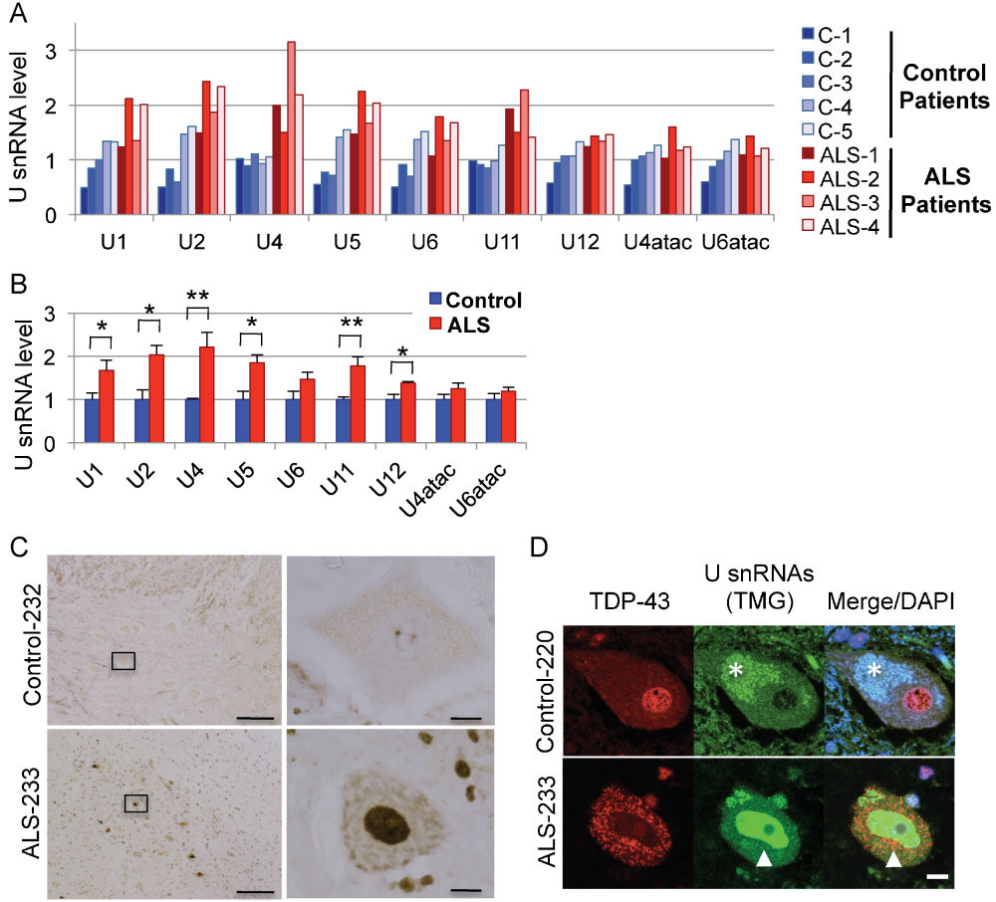
Expression levels of U snRNAs are up-regulated in cervical spinal cords of ALS patients The RNAs were isolated from cervical spinal cords of four ALS patients (ALS-1 to ALS-4) or five control patients with other neurological disease (C-1 to C-5), and U snRNA levels were determined by quantitative RT-PCR as in Fig 4. Detailed clinical information is listed in Supporting Information Table S1.Mean U snRNA levels of control and ALS patients determined in **A** were plotted. Average amounts of U snRNAs from the five control patients were used for normalization. Robust U snRNA misregulation was observed in ALS patients. (Bars: standard errors, *p < 0.05, **p < 0.01, Student's *t*-test).Immunohistochemistry of spinal cords from patients with control disease or ALS using an anti-2,2,7-trimethylguanosine (TMG) antibody, which recognized the 5′ cap structure of snRNA. Boxed areas were shown as the magnified images (Right panels). Bars: 500 µm (left), 20 µm (right).Immunofluorescence staining of spinal motor neurons using anti-TMG and anti-TDP-43 antibodies. Note that strong accumulation of U snRNAs in nucleus of motor neurons from ALS patients (arrowheads). Asterisks show autofluorescence derived from lipofuscin in the cytoplasm. Bars: 10 µm.

Since an abnormal disappearance of TDP-43 from nuclei was observed in motor neurons from sporadic ALS patients and Gems were lost in these neurons (Fig 3) and TDP-43 was important for maintaining the proper integrity of spliceosome U snRNPs (Fig 4), we thought it would be of high interest to investigate whether U snRNA and U snRNP misregulation occurs in affected regions of ALS patients. Frozen cervical spinal cords from four sporadic ALS patients, with spinal cords from five other neurological disease patients serving as controls, were analysed for levels of U snRNAs and other mRNAs. Detailed clinical information is listed in Supporting Information Table S1. Almost all U snRNAs were upregulated in the spinal cords of ALS patients when compared with the control spinal cords (Fig 5A and B). This result confirms that the misregulation of U snRNA levels occurs in the affected region of ALS patients.

We next investigated whether this dysregulation occurs specifically in affected motor neurons in spinal cords of ALS patients. To this end, we stained spinal cords with an anti-2,2,7-trimethylguanosine (TMG) antibody that recognized the 5′ cap structure of U snRNAs. This staining revealed strong accumulations of U snRNAs in motor neurons from ALS patients. TMG staining was higher in motor neuron nuclei from ALS spinal cords than in nuclei from control spinal cords (Fig 5C and D). These results demonstrate that U snRNAs are upregulated in affected motor neurons from ALS patients.

We further asked whether these alternations in U snRNA levels would be seen in the affected regions of patients with other diseases with TDP-43 dysfunction, such as FTLD-TDP. Expression levels of U snRNAs in the temporal lobes of FTLD-TDP patients were analysed by both quantitative RT-PCR and immunohistochemistry using anti-TMG antibody. We found that they were not significantly altered compared with those in control patients (Supporting Information Fig S5A–C). Aberrant strong TMG staining was not observed in neuronal nuclei in FTLD-TDP temporal lobes, despite TDP-43 pathology. Moreover, analyses of long non-coding RNA (lnc RNA) levels demonstrated that NEAT1, which is the most upregulated RNA substrate of TDP-43 in FTLD-TDP affected regions (Tollervey et al, 2011), was not upregulated in ALS spinal cords (Supporting Information Fig S6A–D). These results indicate that the patterns of snRNA/lncRNA dysregulation in neurons with TDP-43 depositions differ among distinct neuronal cell types, and the strong up-regulation of U snRNAs in nuclei is prominent in motor neurons from ALS spinal cords but not in FTLD-TDP temporal lobes.

### Abnormal accumulation of U snRNPs in motor neuron nuclei of ALS spinal cords

**Figure 6:**
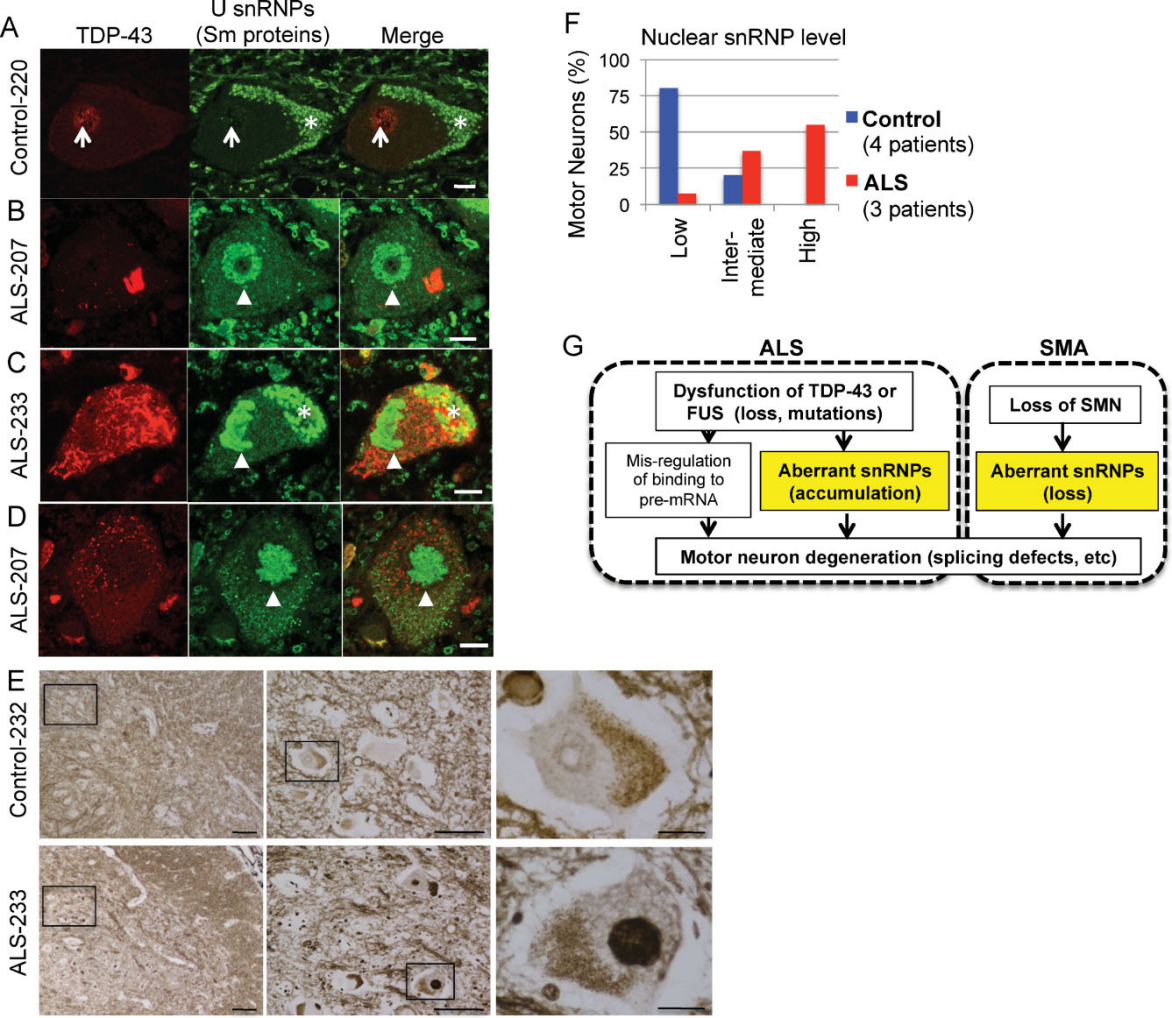
Abnormal accumulation of U snRNPs in motor neuron nuclei of ALS patients **A–D.** Immunofluorescent staining of TDP-43 and U snRNPs using an anti-Sm proteins antibody (Y12) in spinal cord motor neurons from patients with control diseases (**A**) or ALS (**B**–**D**). Arrow shows colocalization of the Y12 antigen and TDP-43 (**A**). The Y12 antigen accumulated in nuclei of ALS motor neurons with TDP-43 mislocalization (**B–D**). Arrowheads (**B–D**) show accumulated U snRNPs in motor neuron nuclei from ALS patients. Asterisks show autofluorescence derived from lipofuscin in the cytoplasm. Bars: 10 µm.**E.** Immunohistochemistry of U snRNPs using an anti-Sm proteins antibody (Y12) in spinal cord motor neurons from patients with control diseases or ALS. Boxed areas in the left and middle panels were shown as the magnified images in the middle and right panels, respectively. Bars: 500 µm (left), 100 µm (middle), 20 µm (right).**F.** Quantification of U snRNP immunofluorescence levels in motor neuron nuclei. Motor neurons from four control patients (blue, *N* = 35) and three ALS patients (red, *N* = 105) were analysed for U snRNP fluorescence intensity in their nuclei. The level of fluorescence intensity of U snRNPs in nuclei compared with cytoplasm were classified as low (nuclear U snRNP level is lower than cytosol), intermediate (nuclear U snRNP level is equal to cytosol), or high (nuclear U snRNP level is higher than cytosol), and plotted. Representative images of motor neurons showing low, intermediate or high nuclear U snRNP levels are shown in Supporting Information Fig S7.**G.** Model for mechanism underlying neurodegeneration in ALS with dysfunction of TDP-43 in comparison to SMA. In this study, we show that TDP-43 localizes in nuclear Gem through association with SMN complex, and is involved in maintenance of spliceosome through controlling the levels of U snRNAs. In ALS patients and SMA mice, U snRNA levels are misregulated in spinal cord. Intriguingly, accumulation of U snRNA is seen in ALS, while loss of U snRNAs is reported in SMA. Moreover, Gems are lost and spliceosomal U snRNPs abnormally accumulate in nuclei of motor neurons from ALS patients. These findings indicate collapse of spliceosome integrity is the critical process common to motor neuron degeneration in ALS and SMA, and may explain cell-type specific vulnerability in motor neurons.

We further investigated whether protein components of U snRNPs were also altered in motor neurons from ALS patients' spinal cords as well as snRNAs, the RNA components of snRNPs. The detailed analysis using immunofluorescent staining with anti-TDP-43 and anti-Sm proteins (Y12) antibodies reveals the accumulation of TDP-43 and snRNPs in the same nuclear body in nuclei of some motor neurons (Fig 6A, arrow) as seen in cultured cells; however, the staining intensity of the anti-Sm proteins (Y12) antibody in nuclei was very weak compared with that seen in cultured cells (Fig 4A). In ALS motor neurons, U snRNPs were extensively accumulated and formed aberrant aggregates in nuclei (Fig 6B–D, and Supporting Information Fig S7), as seen with anti-TMG antibody (Fig 5C and D). Immunohistochemistry also confirmed striking accumulation of U snRNPs in ALS motor neuron nuclei (Fig 6E), but not in nuclei of hippocampal neurons from FTLD-TDP patients (Supporting Information Fig S5D–F). The quantification analysis of the Y12 staining in motor neurons from four control patients and three ALS patients revealed that aberrant U snRNPs accumulation was highly specific to ALS motor neurons (Fig 6F). This abnormal nuclear accumulation of spliceosomal U snRNPs as well as snRNAs could lead to abnormal splicing in ALS motor neurons, resulting in neurodegeneration (Fig 6G).

## DISCUSSION

In this study, we show that TDP-43 localizes to Gems through an association with an SMN/FUS complex and is critically involved in Gem formation and spliceosome maintenance by controlling U snRNA levels. Dysfunction of these proteins impairs the spliceosome resulting in abnormal splicing in motor neurons and neurodegeneration (Fig 6G). We also show that TDP-43 and SMN-dependent spliceosome dysregulation is common to the motor neuron diseases ALS and SMA, respectively, but not FTLD-TDP. In tissue from sporadic ALS patients, or following TDP-43 knockdown in cells, nuclear Gems collapsed and expression levels of U snRNAs spliceosome components were aberrantly up-regulated. Furthermore, U snRNPs aberrantly accumulated in motor neuron nuclei from ALS patients, but not in temporal cortex neurons from FTLD-TDP patients. Our findings strongly indicate that abnormal U snRNP level, which can cause abnormal RNA splicing and/or isoform expression (Berg et al, 2012), is an important mechanism resulting in neurodegeneration common to the motor neuron diseases ALS and SMA (Fig 6G).

The most provocative findings in our study revealed that U snRNA misregulation was observed in several distinct contexts: cells with a decreased expression of TDP-43 (Fig 4B–D) and spinal cord tissue samples of ALS patients (Fig 5), but not in the affected brain regions of FTLD-TDP (Supporting Information Fig S5A and B). Cell type- or tissue- specific alterations in U snRNA repertoires were identified in cells with low levels of SMN and in SMA mouse tissues (Gabanella et al, 2007; Zhang et al, 2008). Similarly, we observed that different sets of U snRNAs were misregulated in neuronal and non-neuronal cells following TDP-43 depletion. Although the direction of U snRNAs misregulation is different between the two diseases; upregulated U snRNAs in ALS, and downregulated U snRNAs in SMA, our work is the first to imply that motor neurons may be sensitive to the collapse of spliceosome, resulting in abnormal splicing through alteration of U snRNP levels (Fig 6). Interestingly, transgenic mice overexpressing poly-Q binding protein-1, which binds to the U5 snRNP component, under a ubiquitous promoter show a late-onset motor neuron disease-like phenotype (Okuda et al, 2003; Waragai et al, 2000). Therefore, motor neurons might be sensitive to snRNP alterations, which would be a potential target for the therapy of motor neuron diseases.

Our results clearly indicate that TDP-43 and SMN might function in a common pathway, namely the regulation of splicing through the maintenance of U snRNP repertoires. Previous studies have demonstrated a genetic link between ALS and SMA. Aberrant copy numbers of SMN1 or SMN2 genes increase the risk of sporadic ALS and disease severity (Andersen & Al-Chalabi, 2011; Blauw et al, 2012; Corcia et al, 2006; Veldink et al, 2001, 2005). Our observation that TDP-43 regulates U snRNAs and associates SMN complex suggests a major function shared between TDP-43 and SMN and supporting a genetic link between ALS and SMA.

Recent studies identifying RNA targets of TDP-43 (Polymenidou et al, 2011; Sephton et al, 2011; Tollervey et al, 2011; Xiao et al, 2011) demonstrated that TDP-43 directly binds to some pre-mRNAs to regulate RNA splicing. However, not all TDP-43 binding sites in pre-mRNAs (as determined by CLIP-seq) are located close to splicing sites, suggesting that TDP-43 might regulate splicing through both direct binding to pre-mRNA and other mechanisms. Our study suggests that TDP-43 also regulates spliceosomal U snRNP biogenesis, which would provide another mechanism of TDP-43-mediated regulation of splicing. Abnormal splicing in motor neurons from ALS patients was reported before (Rabin et al, 2010), however, we are not able to correlate abnormally spliced genes in ALS with abnormal U snRNA repertories at present. The genes indispensable for motor neuron survival may be identified in the future by comparing the numerous abnormally spliced genes reported in SMA with the same genes in ALS.

Although U snRNAs were upregulated only about twofold in the whole spinal cord by quantitative RT-PCR, intense U snRNA staining in ALS motor nuclei indicates much higher U snRNAs upregulation in ALS motor neuron nuclei (presumably more than 100-fold, considering the number of motor neuron much smaller than that of the other spinal cord cell types; Fig 5C and D). Moreover, U snRNAs accumulate and sometimes form aggregates with proteins (Fig 6A–E). Therefore, we speculate that the abnormal accumulation of U snRNPs (they are likely to be non-functional) in ALS motor nuclei could have a substantial impact on RNA splicing and metabolism in motor neurons.

We discovered that TDP-43 and FUS/TLS localizes to nuclear Gems and that Gems are lost in motor neurons of spinal cords from ALS patients as well as TDP-43 depleted or FUS/TLS knockout cells. This is similar to observations in SMA patient-derived cells and SMA mouse models. The number of Gems is correlated with SMA disease severity in fibroblast from SMA patients (Coovert et al, 1997). Furthermore, a recent study showing a reduced number of Gems in the fibroblasts from familial ALS cases with TDP-43 or FUS mutations (Yamazaki et al, 2012) strengthens our findings on the importance of Gem in motor neuron survival. U snRNPs are thought to be stored in Gems for recycling; therefore, TDP-43 might be important for the maintenance of U snRNPs in Gems. The relationship between Gems and TDP-43 has been investigated in several studies. One study demonstrated that the alternatively spliced minor form of mouse TDP-43, which is lacking the C-terminal portion, interacted with SMN (Wang et al, 2002). Another study demonstrated the co-localization of full-length TDP-43 and SMN in human non-neuronal cells (Fiesel et al, 2010), however, additional studies claimed that the colocalization of TDP-43 and SMN was not detected in rat and mouse neurons (Casafont et al, 2009; Shan et al, 2010). In contrast, our study clearly demonstrated that endogenous human TDP-43 was localized in Gems of cultured cells and human motor neurons by coimmunostaining with TDP-43, SMN and Gemin8 (Fig 1A–E and 3). The loss of Gems seen in motor neurons of ALS patients (Fig 3), coupled with the fact that eliminating TDP-43 from mouse neurons *in vivo* leads to the loss of Gems (Shan et al, 2010), clearly supports our findings that TDP-43 and perhaps FUS/TLS is important for Gem formation and U snRNPs biogenesis, as observed before in a similar way with SMN. Furthermore, SMA mutations in the tudor domain of SMN, which is crucial for binding to Sm proteins, abolished SMN association with TDP-43 and FUS/TLS (Fig 2G), supporting an importance of SMN/TDP-43/FUS complex in the biogenesis of spliceosome and in motor neuron degeneration. Moreover, profilin1, which binds to SMN and localizes to Gem (Giesemann et al, 1999), was recently discovered as an ALS causative gene product (Wu et al, 2012). Overexpression of an ALS causing-mutant SOD1 prevents the formation of Gem in the motor neurons of mice (Kariya et al, 2012). Therefore, abnormal Gem formation and/or abnormal U snRNPs formation may underlie the mechanisms of motor neuron degeneration.

The importance of the C-terminal region of TDP-43 was demonstrated through the identification of a domain required for the proper targeting of TDP-43 to Gems and association with SMN (Fig 2A–D), and also by the identification of interactions with several proteins implicated in RNA metabolism (Supporting Information Fig S4). TDP-43 associated with various proteins implicated in RNA metabolism, including proteins involved in pre-mRNA splicing, translational control and the miRNA pathway. Considering that most ALS-linked mutations reside in the C-terminus of TDP-43 (Lagier-Tourenne & Cleveland, 2009), C-terminal region-mediated regulation of RNA metabolism may be disturbed in motor neuron diseases. The proteins we identified could therefore be important to analyse for further potential contributions to motor neuron degeneration.

It is intriguing that TDP-43 localized not only to Gems but also to paraspeckles and nuclear speckles. The long non-coding RNA (lncRNA) NEAT1 (also called Men ε/β) is indispensable for the formation of paraspeckles, where highly edited mRNAs are stored (Bond & Fox, 2009). Furthermore, nuclear speckles are enriched with spliceosomal U snRNPs, other splicing regulators important for RNA splicing such as SR proteins and Malat1 lncRNA (Mao et al, 2011). The expression of NEAT1 and Malat1 lncRNA, both of which have multiple TDP-43 binding sites, is elevated in FTLD-TDP brain (Tollervey et al, 2011). Our study with FTLD-TDP patients also demonstrated an increased expression level of NEAT1 (Supporting Information Fig S6B and D). However, NEAT1 was not significantly altered in ALS spinal cord and TDP-43 depleted cells (Supporting Information Fig S6A and C), suggesting distinct regulations of this lncRNA in different disease conditions. Nevertheless, the enrichment of TDP-43 in paraspeckles and speckles should be examined further to determine any potential role in RNA metabolism in these nuclear subdomains. Taken together, the expression of U snRNA spliceosome components was aberrant and long non-coding RNAs were normal in ALS spinal cords, but these profiles were reversed in FTLD. These results suggest that while neurodegenerative diseases with distinct causal genes (ALS, SMA) can have disruptions in a common biochemical pathway, diseases with the same causal gene (ALS, FTLD) can also have disruptions in distinct pathways.

In conclusion, we show here that TDP-43 and SMN share a common function in spliceosomal U snRNP biogenesis. Dysfunction of these distinct proteins in ALS and SMA leads to collapse of spliceosome integrity and abnormal splicing in motor neurons. We expect that further investigation of defects in RNA metabolism common to these motor neuron diseases but different from a related brain disease should provide explanation to the cell-type specific vulnerability observed in neurodegenerative diseases. In addition, targeting spliceosome and/or Gem stability in motor neurons may represent a new class of candidate therapeutics for motor neuron diseases.

## MATERIALS AND METHODS

### Expression vectors

The open reading frame of human *TARDBP* was inserted into p3XFLAG-CMV14 vector (Sigma), resulting in the insertion of an 18 amino acid spacer between the TDP-43 C-terminus and 3XFLAG peptides. Coding regions of TDP-43 fused with 3xFLAG were subcloned into pF5K-CMV-neo vector (Promega). For FUS/TLS expression, the open reading frame of human *FUS/TLS* fused with 3XFLAG on its N-terminus were subcloned into pF5K-CMV-neo or pF5A-CMV-neo vector (Promega).

### Cell culture and immunofluorescence

Hela cells were maintained in DMEM with high glucose (Gibco) supplemented with l-glutamine, and 10% foetal bovine serum (Gibco). SH-SY5Y cells were maintained in Advanced DMEM/F12 (Gibco) with non-essential amino acids, sodium pyruvate, GlutaMAX (Gibco), and 10% foetal bovine serum. Hippocampal neurons were isolated from E16.5 C57BL6 or *FUS*^*−/−*^ mouse embryos and cultured essentially as described (Huang et al, 2007). Cells were cultured on chamber slides (Lab-Tek, Nunc), fixed with 4% paraformaldehyde for 10 min and permeabilized with 0.1% Triton X-100. For paraspeckle staining with mouse anti-p54 (BD transduction), cells were fixed with cold methanol. Non-specific binding was blocked by incubation with 1% normal goat serum prior to the application of primary antibody. Antibodies used were as follows: rabbit anti-TDP-43 (ProteinTech), anti-coilin (Sigma, clone pδ), mouse anti-p54 (BD transduction), anti-SRSF2 (Sigma, clone SC-35), mouse anti-SMN (BD transduction, 610646), rabbit anti-SMN (Santa Cruz, sc-15320), mouse anti-Gemin8 (Santa Cruz, sc-130669), rabbit anti-FUS/TLS (Abcam, 70381), anti-dimethylated Sm proteins (Lab Vision Corp./Thermo Scientific, clone Y12), anti-TMG (Santa Cruz, clone K121), mouse anti-FLAG (M2), rabbit anti-FLAG (Sigma) and rabbit anti-GFP (MBL).

### Knockdown of protein expression in cells

To eliminate TDP-43 expression, Hela cells were transfected with 4 nM Stealth siRNA for *TARDBP* (Invitrogen, ID#HSS177422 or originally designed oligos with the sequences listed in Supporting Information Table S2) or control siRNA (Invitrogen, LoGC#2 or #3) in suspension at 1.5 × 10^5^ cells/ml using Lipofectamine RNAiMax (Invitrogen). After an overnight culture, cells were then transfected with siRNA once more, and then cultured for two more days. For SH-SY5Y cells, cells were transfected in suspension at 3 × 10^5^ cells/ml. After 3 days of culture, cells were divided into three dishes, transfected with siRNAs again and cultured for three more days.

### Immunoprecipitation

Cells were transfected with pF5K-TDP-43-3xFlag constructs using X-tremeGENE HP (Roche). Cells were harvested and washed with PBS 3 times. TBS supplemented with 0.2% Triton X-100, protease inhibitors (Nakalai, Japan), and RNase inhibitor SUPERase-In (Ambion) was used as a Lysis buffer. The cell pellet was then lysed in the same volume of Lysis buffer on ice for 10 min. The nuclear membrane was disrupted by passage through a 28 G needle and then centrifuged at 14,000*g* for 15 min. Supernatants were collected as total cell extracts. After the protein concentration of cell extracts was adjusted to 5–8 mg/ml with Lysis buffer, cell extracts were mixed with agarose beads conjugated with anti-FLAG antibody (M2-agarose, Sigma) and incubated overnight at 4°C. After washing with Lysis buffer for three times, non-specific protein binding to the anti-FLAG agarose beads was washed out by incubating with FLAG peptide at 50 µg/ml for 15 min at 4°C. This step was critical to wash out non-specific or weak binding to the anti-FLAG agarose beads. To elute the protein complex with 3XFLAG-tagged protein, 3XFLAG peptides were added to the agarose beads at 500 µg/ml and incubated for 1 h at 4°C. Eluted proteins were then analysed by immunoblotting. For immunoprecipitation of HA-tagged protein, anti-HA-agarose (Sigma) was used instead of anti-FLAG agarose, and the precipitated proteins were eluted with SDS-sample buffer.

For immunoprecipitation of mature U snRNPs, a nuclear pellet was obtained from suspension cells using Buffer A (50 mM Hepes pH 7.5/1 mM MgCl_2_/1 mM EGTA/1 mM DTT/protease inhibitors) on ice for 10 min followed by centrifugation at 3000*g* for 5 min. The nuclear pellet was lysed in Buffer A supplemented with 150 mM KCl, 1% NP-40, 10% glycerol, and RNase inhibitor SUPERase-In (Ambion) and then the nuclear membrane was disrupted by passage through a 27 G syringe 10 times and a repeated freeze/thaw cycle 3 times. Nuclear extract was obtained after the removal of cell debris by centrifugation at 20,000*g* for 10 min. Anti-Sm proteins monoclonal antibody (Y12) and mouse immunoglobulin (as negative control) were used for immunoprecipitation. Antibodies used for Western blot were as follows: mouse anti-FUS/TLS (Santa Cruz, sc-47711), rat anti-PRPF3 (MBL, clone 4E3), goat anti-U1-70K (Santa Cruz), mouse anti-PABP (Sigma, clone 10E10) and rabbit anti-eIF4G (Cell Signaling, #2498).

**The paper explained**

PROBLEM:

The motor neuron diseases (ALS) and spinal muscular atrophy (SMA) are caused by dysfunction of proteins involved in RNA metabolism. For ALS, the RNA-binding proteins TDP-43 and FUS/TLS, have been implicated, while in SMA the protein SMN, essential for biogenesis of spliceosomal component snRNPs, is critical. A key question is whether there is a shared defective mechanism in RNA metabolism common to these two diseases.

RESULT:

We report a convergent function for TDP-43, FUS/TLS and SMN by showing that the genes for these diseases share a common mechanism: maintenance of nuclear Gems and controlling the level of U snRNA spliceosomal complex. In ALS spinal motor neurons as well as TDP-43 depleted neurons, we observed disruption of Gems and abnormal accumulation of U snRNAs/snRNPs. Together, our findings indicate that TDP-43, FUS/TLS and SMN are important for spliceosome integrity, and that collapse of the spliceosome is the critical mechanism that must underlie the neurodegenerative process in both ALS and SMA.

IMPACT:

Our study reveals the important role of nuclear Gems and spliceosomal U snRNPs in motor neuron survival. Although it requires more investigation, our work substantially contributes to understanding the molecular mechanism of motor neuron disease by providing evidence linking for the first time the selective vulnerability of motor neurons to spliceosome breakdown in Gems of ALS and SMA. Furthermore, targeting spliceosome and/or Gem stability in motor neurons may represent a new class of candidate therapeutics for motor neuron diseases.

### FUS/TLS knockout mice

ES cell clone (HMA274) with β-Gal-neo cassette inserted between exon 2 and 3 of *FUS/TLS* gene were obtained from mutant mouse regional resource centers at University of California, Davis, and used to generate FUS/TLS heterozygous mice with support by Research Resource Center of RIKEN Brain Science Institute. Genotyping of mice with disrupted FUS/TLS allele was performed using RT-PCR, and FUS/TLS protein levels were confirmed by Western blot analysis. Heterozygote mice (F3) were intercrossed to generate *FUS*^−/−^ mice.

### Postmortem human tissues

Specimens of spinal cords from five patients with sporadic ALS and seven other neurological disease patients as controls, as well as temporal lobes from three patients with FTLD-TDP and four other neurological disease patients as controls, were obtained by autopsy with informed consent (Supporting Information Table S1). The diagnosis of ALS was confirmed by El Escorial diagnostic criteria as defined by the World Federation of Neurology and with the presence of TDP-43 pathology, as detected by histopathology. For the diagnosis of FTLD-TDP, selective sections were immunostained with antibodies against phosphorylated tau, ubiquitin, phosphorylated TDP-43 and FUS/TLS to select FTLD-TDP (Cairns et al, 2007). All patients with ALS and FTLD-TDP showed no hereditary traits. The collection of tissues and their use in this study was approved by the ethics committee of Nagoya University Graduate School of Medicine, Fukushimura Hospital, Tokyo Metropolitan Institute, and RIKEN. Tissues for RNA analysis were immediately frozen using liquid nitrogen and stored at −80°C until use. For immunofluorescent staining, 6 µm sections were prepared from paraffin-embedded tissues, deparaffinized, microwaved for 20 min in 50 mM citrate buffer (pH 6.0), treated with TNB blocking buffer (PerkinElmer, Boston, MA) and then incubated with primary antibodies. After washing, sections were incubated with Alexa-546-conjugated goat anti-rabbit IgG (1:1000; Invitrogen) and Alexa-488-conjugated goat anti-mouse IgG (1:1000; Invitrogen) for 30 min, mounted with Prolong gold antifade reagent (Invitrogen) and then imaged with a laser confocal microscope (LSM710, Carl Zeiss, Jena, Germany). The position of the nucleus was confirmed by TO-PRO-3 Iodidel (Invitorgen). For immunohistochemistry, sections were depareffinized and boiled for 20 min in 50 mM citrate buffer (pH 6.0), treated with 3% goat serum/0.5% tween-20/PBS supplemented with Avidin solution (Vector, Avidin/Biotin Blocking kit, #SP-2001), and then incubated with primary antibodies in 3% goat serum/0.5% tween-20/PBS supplemented with Biotin solution (Vector, Avidin/Biotin Blocking kit). After washing, sections were incubated with Biotin-conjugated anti-mouse IgG or anti-rabbit IgG (1:400, Vector) in 0.05%Tween-20/PBS. Signals were visualized with Vectastain ABC kit (Elite, #PK-6100) and Metal Enhanced DAB substrate kit (Thermo Scientific, #34065).

### Quantitative RT-PCR

Prior to RNA extraction, cultured cells were harvested and stored in RNAlater (Ambion), and frozen tissue samples were stored in RNAlater ICE (Ambion). Total RNA containing a small RNA fraction was extracted with a *mir*Vana miRNA Isolation Kit (Ambion) according to the manufacturer's instructions, and then treated with DNase (TURBO DNA-free Kit, Ambion) for either 20 min or 1 h depending on whether the source was cultured cells or tissue samples, respectively. U snRNAs were transcribed with specific primers as described previously (Zhang et al, 2008), and RNA levels were quantified with specific primers as described previously (Zhang et al, 2008) using the Syber Green system (Applied Biosystems). The primers we used are listed in Supporting Information Table S3. All PCR reactions were performed in triplicate. RNA levels in samples were normalized with GAPDH for mRNA, and average of 5S and 5.8S for small RNA.

For more detailed Materials and Methods see the Supporting Information.

## Author contributions

HT and KY designed the study; HT, YI, AF and AK performed the experiments; YI, HH, NA, FT, YH, HA, SM and GS obtained the patient autopsy samples and performed neuropathological and clinical diagnosis; HH and SM advised the staining of human sample; YI, NA, FT and GS provided critical inputs for the manuscript; HT analysed the data and KY provided inputs to analysis; HT and KY wrote the manuscript. All authors approved the manuscript.

## Supplementary material

As a service to our authors and readers, this journal provides supporting information supplied by the authors. Such materials are peer reviewed and may be re-organized for online delivery, but are not copy-edited or typeset. Technical support issues arising from supporting information (other than missing files) should be addressed to the authors.
