# Supplementary material for: Spliceosome integrity is defective in the motor neuron diseases ALS and SMA
Source: EMBO Mol Med. 2013 Jan 25;5(2):221–34. doi: 10.1002/emmm.201202303 (PMC3569639; doi:10.1002/emmm.201202303)
Supplement: Supplementary file 1 [file emmm0005-0221-SD1.pdf]

Spliceosome Integrity is Defective in the Motor Neuron Diseases ALS and SMA

Hitomi Tsuiji, Yohei Iguchi, Asako Furuya, Ayane Kataoka, Hiroyuki Hatsuta, Naoki Atsuta, Fumiaki Tanaka, Yoshio Hashizume, Hiroyasu Akatsu, Shigeo Murayama, Gen Sobue, and Koji Yamanaka

Corresponding author: Koji Yamanaka, RIKEN Brain Science Institute

Review timeline:

Submission date:	28 November 2012
Accepted:	07 December 2012

Transaction Report:

No Peer Review Process File is available with this article, as the authors have chosen not to make the review process public in this case.
