# Supplementary material for: Spliceosome integrity is defective in the motor neuron diseases ALS and SMA
Source: EMBO Mol Med. 2013 Jan 25;5(2):221–34. doi: 10.1002/emmm.201202303 (PMC3569639; doi:10.1002/emmm.201202303)
Supplement: Supplementary file 2 [file emmm0005-0221-SD2.pdf]

Supporting Information

Spliceosome Integrity is Defective in the Motor Neuron Diseases ALS and SMA

Hitomi Tsuiji, Yohei Iguchi, Asako Furuya, Ayane Kataoka, Hiroyuki Hatsuta, Naoki Atsuta, Fumiaki Tanaka, Yoshio Hashizume, Hiroyasu Akatsu, Shigeo Murayama, Gen Sobue, and Koji Yamanaka

Supporting Information Figures

Figure S1: TDP-43 localizes in paraspeckles and speckles, but not in nucleolus, PML body, or SAM68 bodies

Figure S2: Depletion of TDP-43 or FUS

Figure S3: The latter half of C-terminal glycine-rich region of TDP-43 is important for the localization to gems

Figure S4: Identification of TDP-43-interacting proteins reveals that TDP-43 is associated with SMN complex, spliceosomal proteins, and proteins involved in translational control or miRNA processing

Figure S5: Expression levels of U snRNAs in temporal lobes of FTLD-TDP patients.

Figure S6: Expression level of long non-coding RNA or U snRNA in TDP-43-depleted cells and in affected tissues of ALS or FTLD-TDP patients.

Figure S7: Aberrant accumulation of U snRNPs in motor neuron nuclei from ALS patients.

Supporting Information Methods and Tables

Supporting Methods

Table S1: Summary of clinical information of patients with ALS, FTLD-TDP or other diseases

Table S2: Detailed list of proteins identified as TDP-43 binding partner

Table S3: Oligonucleotides used in this study

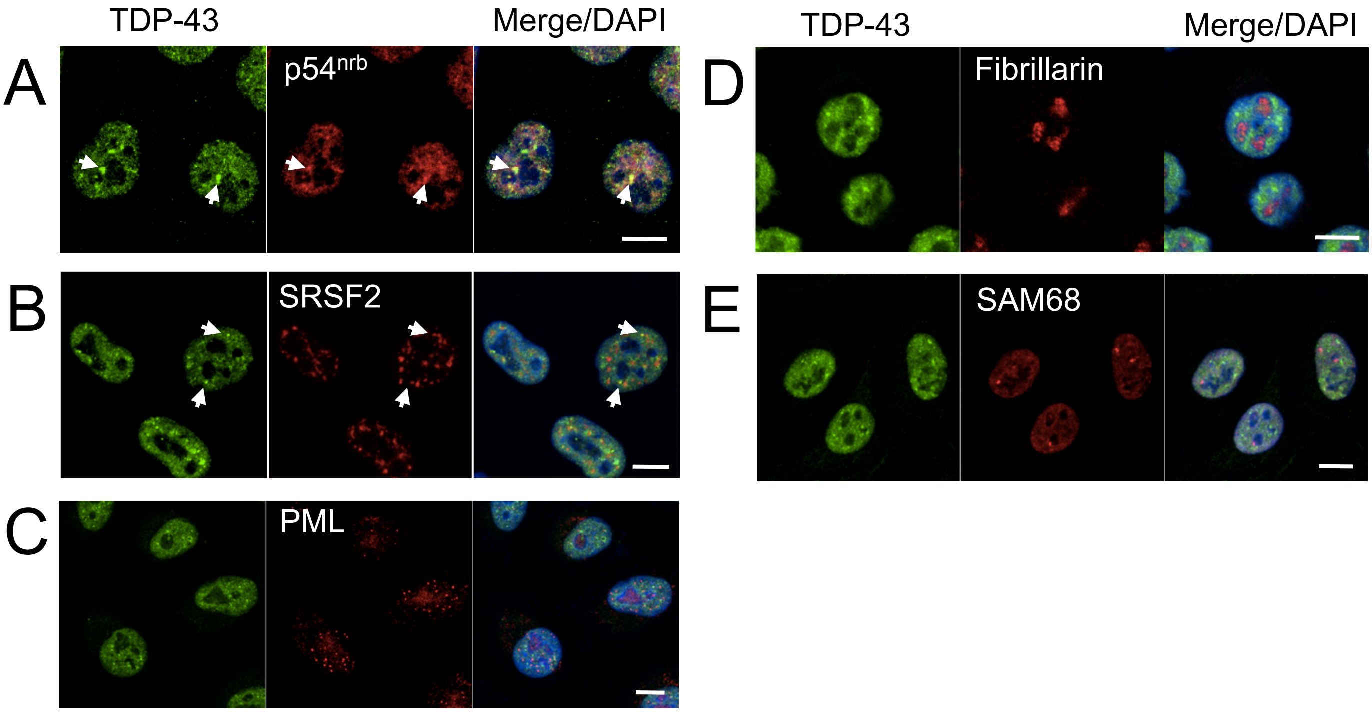

Figure S1. TDP-43 localizes in paraspeckles and speckles, but not in nucleolus, PML body, or SAM68 bodies

A-E. Hela cells were immunostained with the antibodies for TDP-43 and nuclear domain markers, followed by DAPI staining. **A.** Staining of TDP-43 and $p54^{nrb}$. $p54^{nrb}$-accumulated foci indicated paraspeckles, where TDP-43 was accumulated (arrows). **B.** Staining of TDP-43 and SC35/SRSF2. SC35/SRSF2 localized in nuclear speckles, where some TDP-43 bodies were included (arrows). **C.** Staining of TDP-43 and PML. **D.** Staining of TDP-43 and Fibrillarin, nucleolus marker. **E.** Staining of TDP-43 and SAM68. Bars; 10 μm.

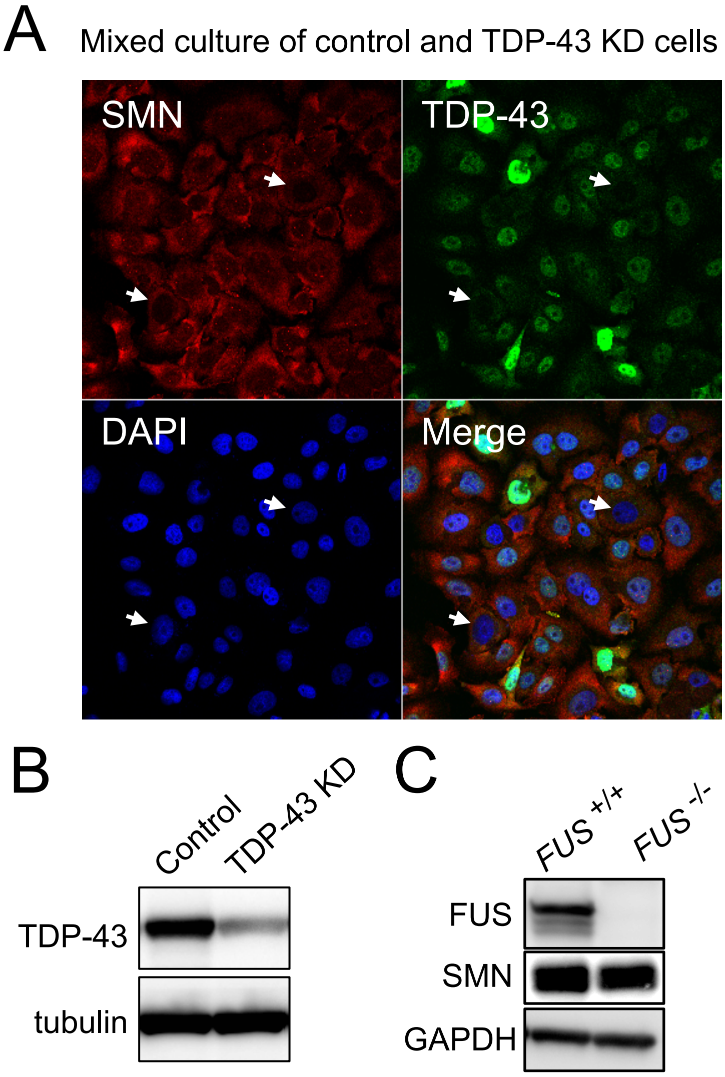

Figure S2. Depletion of TDP-43 or FUS

A. Hela cells treated with siRNAs for TDP-43 or control sequence for 2 days were mixed and stained for SMN and TDP-43. Cells treated with control siRNA show strong TDP-43 expression whereas cells treated with TDP-43 siRNA show various level of TDP-43. Note that cells with no detectable TDP-43 lost gems (arrows). **B.** Western blot analysis of Hela cells treated with siRNAs for TDP-43 or control sequence. **C.** Western blot analysis of brain tissues from $FUS^{-/-}$ mice. The blots confirmed complete elimination of FUS/TLS protein in $FUS^{-/-}$ brain while the level of SMN protein was unaffected.

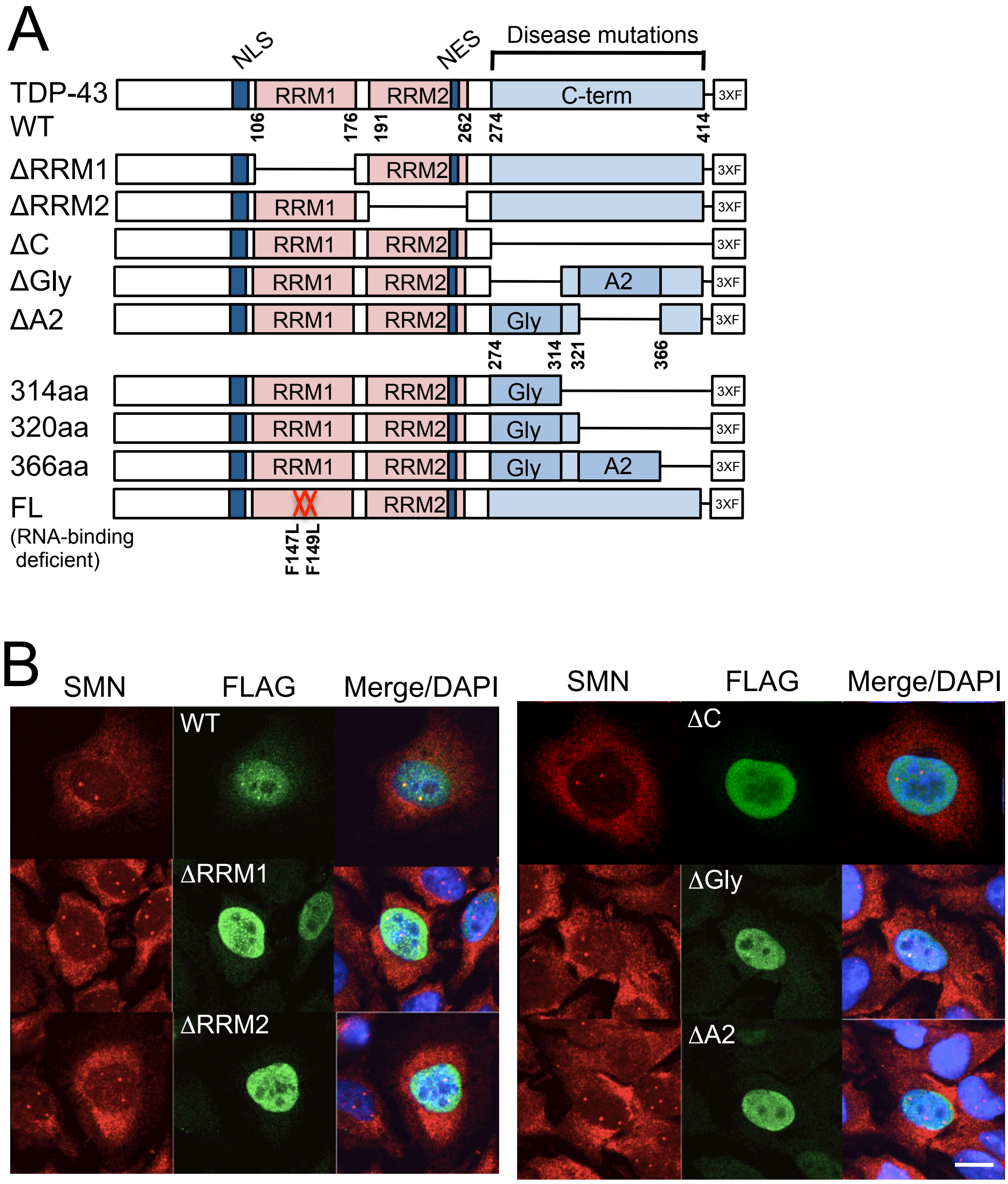

Figure S3. The latter half of C-terminal glycine-rich region of TDP-43 is important for the localization to gems.

A. A schematic diagram of C-terminal 3XFLAG-tagged expression constructs for TDP-43 and its deletion mutants used in this study. **B.** Hela cells were transfected with TDP-43-3XFLAG and its mutants, and stained with anti-SMN and anti-FLAG antibodies. Bar: 10μm. Note that TDP-43 ΔC mutant lost its localization to Gems in nucleus.

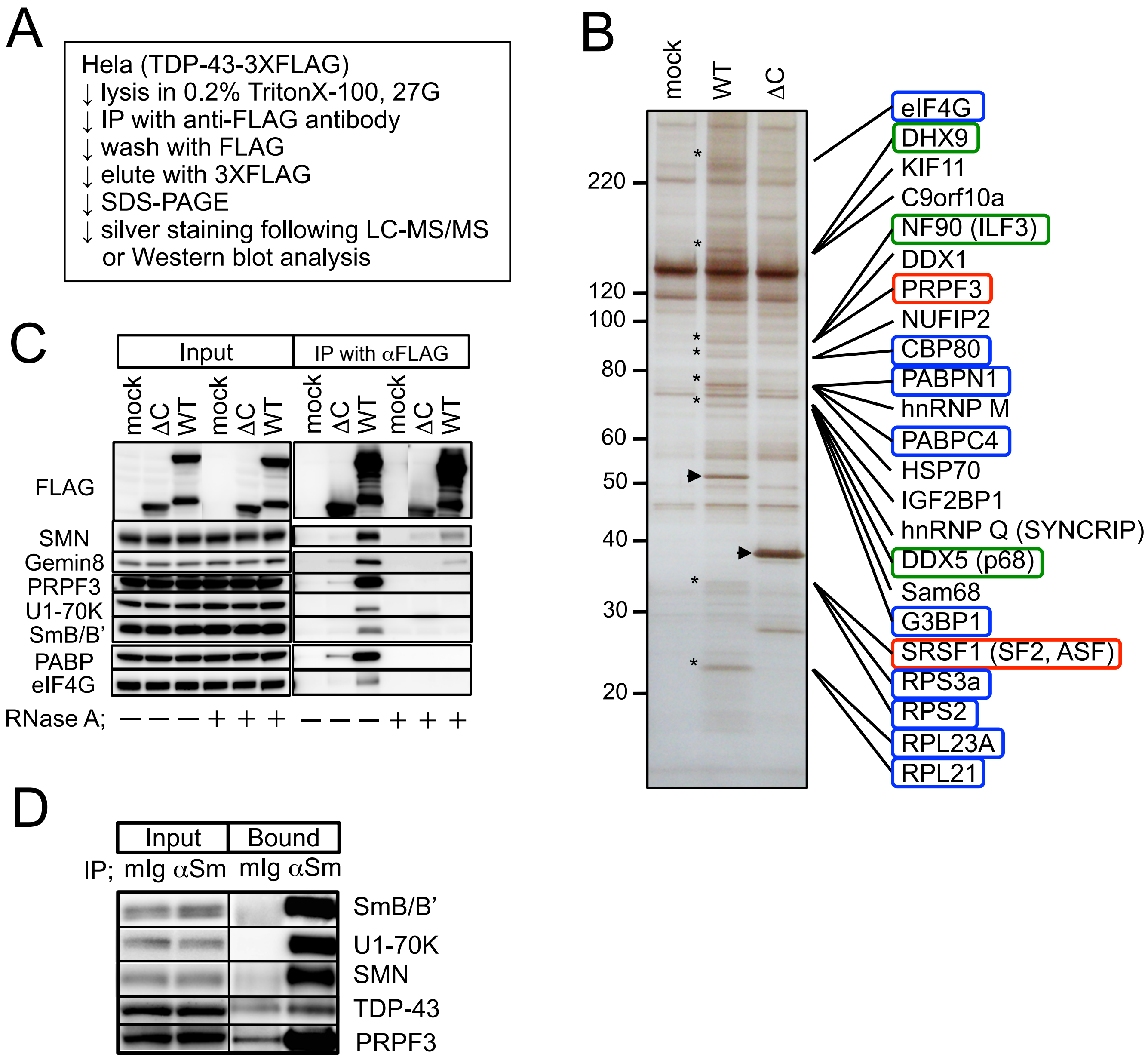

Figure S4. Identification of TDP-43-interacting proteins reveals that TDP-43 is associated with SMN complex, spliceosomal proteins, and proteins involved in translational control or miRNA processing
A. The method for identification of TDP-43-interacting proteins. TDP-43 binding proteins were purified using anti-FLAG antibody from Hela cells transfected with TDP-43-3XFLAG constructs. Immunoprecipitated proteins were subjected to western blot analysis, or separated on SDS-PAGE, and identified by LC-MS/MS following silver staining. **B.** Identification of TDP-43-C-terminus interacting proteins. TDP-43-3xFLAG mutants were expressed in Hela cells, and immunopurified proteins with anti-Flag antibodies were visualized with sliver staining as described in **A**. Representative bands (*) from the purified complex with wild type TDP-43 but not with ΔC TDP-43 were isolated for the determination of proteins by LC-MS/MS. Identified protein were listed and classified into four groups according to their major function in RNA metabolism. (Green: miRNA processing; Blue: translational control; Red: splicing regulation; Not colored: others) **C.** Protein interaction with TDP-43 was dependent on its-C-terminus and RNA binding. TDP-43 interacting proteins were immunoprecipitated with anti-FLAG antibody as described in **A**. Bound fractions were immunoblotted with the specific antibodies as indicated. To test the requirement of RNA binding for their interactions, lysates were treated with or without RNase A for 2 hours before immunoprecipitation. **D.** U snRNPs were immunopurified from nuclear extracts of Hela cells using an anti-dimethylated Sm protein antibody (Y12) (αSm) or control mouse IgG (mIg), and major protein components of U snRNPs or TDP-43 were detected by immunoblotting.

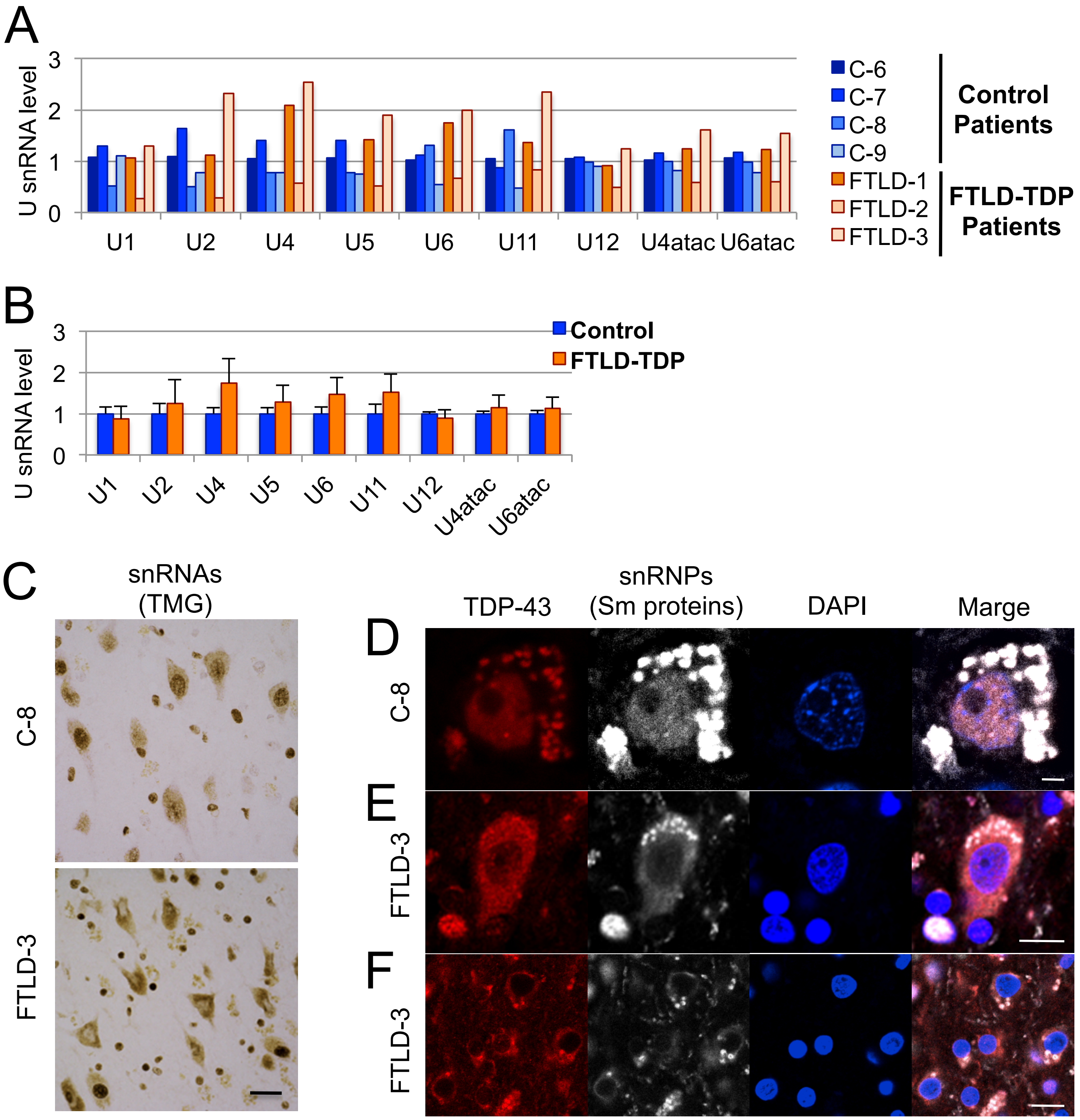

Figure S5. Expression levels of U snRNAs in temporal lobes of FTLD-TDP patients.

A. RNA was isolated from temporal lobes of three patients with FTLD-TDP (FTLD-1 to FTLD-3) or four control patients (C-6 to C-9), and U snRNA levels were determined by quantitative RT-PCR as in Figure 4 and 5. **B.** Mean U snRNA levels of control and FTLD-TDP patients determined in Figure S5A were plotted. Average amounts of U snRNAs of the four control patients were used for normalization. Bars: standard errors, Student's *t*-test revealed no statistical significance.

C. Immunohistochemistry of CA3 region of control or FTLD-TDP temporal lobes with anti-TMG antibody. Bar: 200 µm. **D-F.** Immunofluorescent staining of control **(D)** or FTLD-TDP **(E, F)** temporal lobes with anti-Sm protein antibody (Y12) and anti-TDP-43 antibody. Pyramidal neurons in CA3 **(D, E)** and granule cells in hippocampus **(F)** were shown. Note that snRNAs and snRNPs were more abundant in the cytoplasm than in the nuclei of pyramidal neurons in the CA3 region and granule cells in the dentate gyrus region of the hippocampus, indicating that snRNAs accumulate with TDP-43 in the cytoplasm of degenerated neurons in the temporal lobe. Bars: 5 µm.

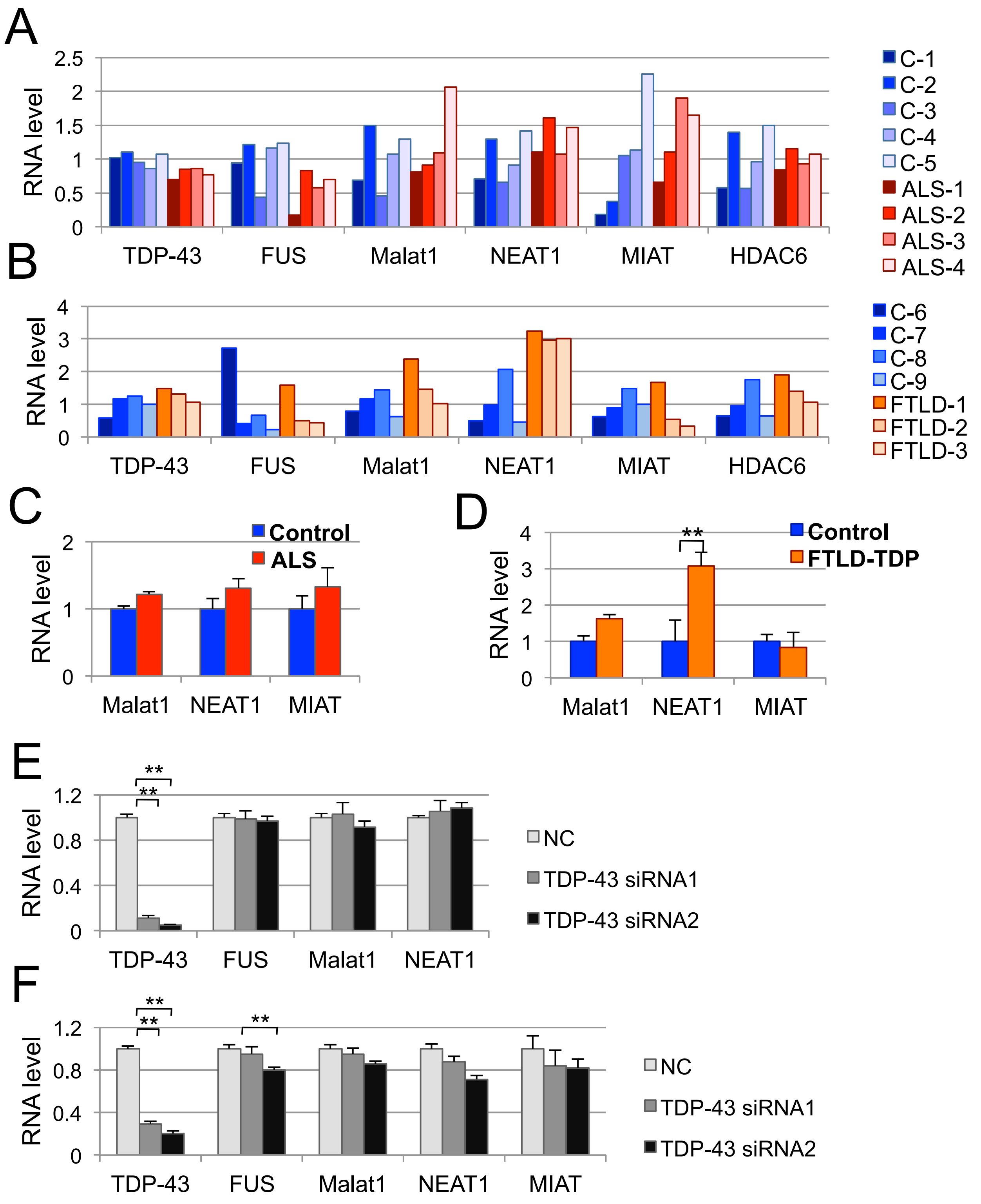

Figure S6. Expression levels of long non-coding RNA or U snRNA in TDP-43-depleted cells and in affected tissues of ALS or FTLD-TDP patients. **A.** Levels of mRNA and long non-coding RNA (lncRNA) in individual patients with ALS (ALS-1 to ALS-4) or disease control (C-1 to C-5). **B.** Levels of mRNA and lncRNA in individual patients with FTLD-TDP (FTLD-1 to FTLD-3) or disease control (C-6 to C-9). **C.** Mean lncRNA levels of control and ALS patients determined in Fig. S6A were plotted. Average amounts of RNAs of the five control patients were used for normalization. **D.** Mean lncRNA levels of control and FTLD patients determined in Fig. S6B were plotted. Average amounts of RNAs of the four control patients were used for normalization. **E, F.** mRNA and lncRNA levels in Hela cells **(E)** or SH-SY5Y cells **(F)** treated with siRNAs for TDP-43 or control were determined by quantitative RT-PCR. Average from 3 independent experiments with triplicate transfections were plotted. Same RNA extracts were used to determine U snRNA levels in Figure 4B and 4C. Bars: Standard Errors, * : p <0.05, **: p <0.01, student t-test.

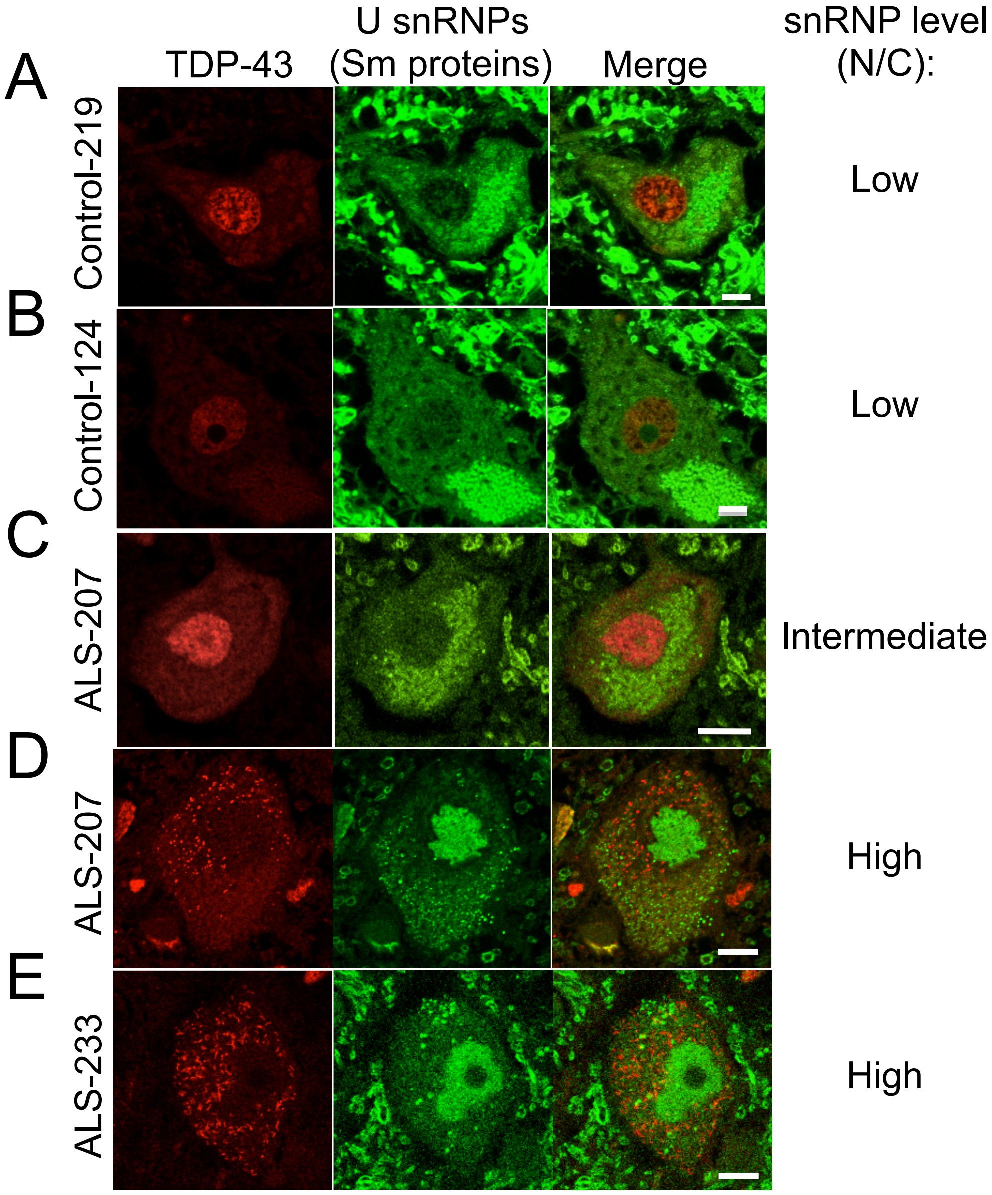

Figure S7. Aberrant accumulation of U snRNPs in motor neuron nuclei from ALS patients. A-E. Staining of TDP-43 and U snRNPs in motor neuron nuclei from control patients and ALS patients. Motor neurons with lower staining of U snRNPs in nucleoplasm than in cytosol were categorized in Low; with higher staining are categorized in High; with similar intensity of U snRNPs staining are categorized in Intermediate. Bars: 10 µm. **(A, B)** Motor neurons from control patients. **(C-E)** Motor neurons from ALS patients. Detailed clinical information is listed in Table S1.

Supporting Information Methods

Immunoprecipitation and identification of proteins by LC-MS/MS

Cells were transfected with pF5K-TDP-43-3xFLAG constructs with X-tremeGENE HP (Roche). Immunopurification of TDP-43 interacting proteins from lysates using anti-FLAG agarose and 3XFLAG peptides were carried out according to the procedures described in Online Methods. Eluted proteins were separated on SDS-PAGE, visualized with silver staining (ProteoSilver Silver Stain Kit, Sigma-Aldrich), and extracted from a gel. Extracted and digested peptides with trypsin were analyzed and identified by LC-MS/MS at RIKEN Brain Science Institute Research Resources Center.

Supporting Information Tables

Table S1. Summary of clinical information of patients with ALS, FTLD-TDP or other diseases

Patient ID	Diagnosis	Age & Gender	Affected /analyzed region	Postmortem time before autopsy (h)
Control-216 (C-1)	Myasthenia gravis	84M	Spinal cord	N/A
Control-218 (C-2)	Parkinson's disease	82M	Spinal cord	N/A
Control-219 (C-3)	Parkinson's disease	68M	Spinal cord	4.5
Control-232 (C-4)	Parkinson's disease	80F	Spinal cord	13
Control-212 (C-5)	Hypertrophic pachymenigitis	70F	Spinal cord	4
Control-124	Malignant lymphoma	67M	Spinal cord	9
Control-220	Malignant lymphoma	76F	Spinal cord	N/A
ALS-208 (ALS-1)	ALS	73M	Spinal cord	3
ALS-230 (ALS-2)	ALS	61F	Spinal cord	4
ALS-231 (ALS-3)	ALS	66M	Spinal cord	N/A
ALS-233 (ALS-4)	ALS	53F	Spinal cord	10
ALS-207	ALS	81F	Spinal cord	2.5
C-6	PA	79F	Temporal lobe	3.5
C-7	PA	78M	Temporal lobe	3
C-8	PA	79M	Temporal lobe	24
C-9	PA	63M	Temporal lobe	4
FTLD-1	FTLD-TDP	79F	Temporal lobe	N/A
FTLD-2	FTLD-TDP	78M	Temporal lobe	N/A
FTLD-3	FTLD-TDP	79M	Temporal lobe	N/A

N/A, not available; M, male; F, female.; PA, physiological aging

Table S2. Detailed list of proteins identified as TDP-43 binding partner

Gene Symble	Gene Name	Number of fragments identifed	Mascot score
eIF4G	eukaryotic translation initiation factor 4 gamma,1	4	190
DHX9 (RHA)	DEAH (Asp-Glu-Ala-His) box polypeptide 9	20	1119
KIF11	Kinesin family member 11	6	303
C9orf10a	Chromosome 9 open reading frame 10	3	229
NF90 (ILF3)	interleukin enhancer binding factor 3	7	409
DDX1	DEAD (Asp-Glu-Ala-Asp) box polypeptide 1	2	130
PRPF3	PRP3 pre-mRNA processing factor 3 homolog	2	102
NUFIP2	nuclear fragile X mental retardation protein interacting protein 2	4	265
CBP80	nuclear cap binding protein subunit 1, 80kDa	4	230
PABPN1	poly(A) binding protein, nuclear 1	21	1216
hnRNP M	heterogeneous nuclear ribonucleoprotein M	14	664
PABPC4	poly(A) binding protein, cytoplasmic 4	10	545
HSP70	heat shock 70kDa protein 8	4	268
IGF2BP1	insulin-like growth factor 2 mRNA binding protein 1	8	486
hnRNP Q (SYNCRIP)	synaptotagmin binding, cytoplasmic RNA interacting protein	5	294
DDX5 (p68)	DEAD (Asp-Glu-Ala-Asp) box polypeptide 5	3	208
Sam68	KH domain containing, RNA binding, signal transduction associated 1	2	131
G3BP1	GTPase activating protein (SH3 domain) binding protein 1	2	129
SRSF1 (SF2, ASF)	serine/arginine-rich splicing factor 1	5	262
RPS3a	ribosomal protein S3A	14	661
RPS2	ribosomal protein S2	4	226
RPL23A	ribosomal protein L23A	3	181
RPL21	ribosomal protein L21	3	153

Table S3. Oligonuceotides used in this study

Oligonucleotide	Sequence
U1 snRNA Fw	GATACCATGATCACGAAGGTGGTT
U1 snRNA Rv	CACAAATTATGCAGTCGAGTTTCC
U2 snRNA Fw	TTTGGCTAAGATCAAGTGTAGTATCTGTTC
U2 snRNA Rv	AATCCATTTAATATATTGTCCTCGGATAGA
U4 snRNA Fw	GCGCGATTATTGCTAATTGAAA
U4 snRNA Rv	AAAAATTGCCAATGCCGACTA
U5 snRNA Fw	GGTTTCTCTTCAGATCGCATAAATC
U5 snRNA Rv	CTCAAAAAATTGGGTTAAGACTCAGA
U6 snRNA Fw	GCTTCGGCAGCACATATACTAAAAT
U6 snRNA Rv	ACGAATTTGCGTGTCATCCTT
U11 snRNA Fw	GTGCGGAATCGACATCAAGAG
U11 snRNA Rv	CGCCGGGACCAACGAT
U12 snRNA Fw	AACTTATGAGTAAGGAAAATAACGATTCG
U12 snRNA Rv	CGACCTTTACCCGCTCAAAA
U4atac snRNA Fw	GCGCATAGTGAGGGCAGTACT
U4atac snRNA Rv	GCACCAAAATAAAGCAAAAGCTCTA
U6atac snRNA Fw	AGGTTAGCACTCCCCTTGACAA
U6atac snRNA Rv	TGGCAATGCCTTAACCGTATG
5S rRNA Fw	CGGCCATACCACCCTGAAC
5S rRNA Rv	GCGGTCTCCCATCCAAGTAC
5.8S rRNA Fw	CGGCTCGTGCGTCGAT
5.8S rRNA Rv	CCGCAAGTGCGTTCGAA
GAPDH Fw	ATGGGGAAGGTGAAGGTCG
GAPDH Rv	GGGGTCATTGATGGCAACAATA
TDP-43 Fw	CCATTGAAATACCATCGGAAGAC
TDP-43 Rv	CCTGGAAACTGGGCTGTAACC
FUS Fw	ATTTGACCATGGTGGAGAGC
FUS Rv	AAGCCCTCTGAGTACAGGCA
Malat1 Fw	CCAGTTTTCCGAGAACCAAA
Malat1 Rv	ATGCTGATCTGCTGCGTATG

NEAT1 Fw	TGGGGGAGTTTCGTACTGAG
NEAT1 Rv	TCTCCAGGACTTGGCAGTCT
MIAT Fw	CATTCTTCCTCCGTCTCAGC
MIAT Rv	GAGAAAAGGAAGAACGCACG
Stealth dsRNA for TDP-43 Fw	CCAACACACUACAAUUGAUAUCAAA
Stealth dsRNA for TDP-43 Rv	UUUGAUAUCAAUUGUAGUGUGUUGG
